# SLOctolyzer: Fully Automatic Analysis Toolkit for Segmentation and Feature Extracting in Scanning Laser Ophthalmoscopy Images

**DOI:** 10.1167/tvst.13.11.7

**Published:** 2024-11-08

**Authors:** Jamie Burke, Samuel Gibbon, Justin Engelmann, Adam Threlfall, Ylenia Giarratano, Charlene Hamid, Stuart King, Ian J. C. MacCormick, Thomas J. MacGillivray

**Affiliations:** 1 School of Mathematics, University of Edinburgh, Edinburgh, UK; 2 Robert O Curle Ophthalmology Suite, Institute for Regeneration and Repair, University of Edinburgh, UK; 3 Centre for Medical Informatics, University of Edinburgh, Edinburgh, UK; 4 School of Informatics, University of Edinburgh, Edinburgh, UK; 5 Clinical Research Facility and Imaging, University of Edinburgh, Edinburgh, UK; 6 Institute for Adaptive and Neural Computation, School of Informatics, University of Edinburgh, Edinburgh, UK; 7 Centre for Clinical Brain Sciences, University of Edinburgh, Edinburgh, UK

**Keywords:** scanning laser ophthalmoscopy (SLO), optical coherence tomography (OCT), retina, image analysis, artificial intelligence

## Abstract

**Purpose:**

The purpose of this study was to introduce SLOctolyzer: an open-source analysis toolkit for en face retinal vessels in infrared reflectance scanning laser ophthalmoscopy (SLO) images.

**Methods:**

SLOctolyzer includes two main modules: segmentation and measurement. The segmentation module uses deep learning methods to delineate retinal anatomy, and detects the fovea and optic disc, whereas the measurement module quantifies the complexity, density, tortuosity, and caliber of the segmented retinal vessels. We evaluated the segmentation module using unseen data and measured its reproducibility.

**Results:**

SLOctolyzer's segmentation module performed well against unseen internal test data (Dice for all-vessels = 0.91; arteries = 0.84; veins = 0.85; optic disc = 0.94; and fovea = 0.88). External validation against severe retinal pathology showed decreased performance (Dice for arteries = 0.72; veins = 0.75; and optic disc = 0.90). SLOctolyzer had good reproducibility (mean difference for fractal dimension = −0.001; density = −0.0003; caliber = −0.32 microns; and tortuosity density = 0.001). SLOctolyzer can process a 768 × 768 pixel macula-centered SLO image in under 20 seconds and a disc-centered SLO image in under 30 seconds using a laptop CPU.

**Conclusions:**

To our knowledge, SLOctolyzer is the first open-source tool to convert raw SLO images into reproducible and clinically meaningful retinal vascular parameters. It requires no specialist knowledge or proprietary software, and allows manual correction of segmentations and re-computing of vascular metrics. SLOctolyzer is freely available at https://github.com/jaburke166/SLOctolyzer.

**Translational Relevance:**

SLO images are captured simultaneous to optical coherence tomography (OCT), and we believe SLOctolyzer will be useful for extracting retinal vascular measurements from large OCT image sets and linking them to ocular or systemic diseases.

## Introduction

The retina is a highly vascularized light-sensitive tissue at the back of the eye. The retinal microvasculature branches from the optic nerve head along the inner surface of the retina and is commonly imaged using color fundus photography, which typically covers a 35- to 50-degree field of view around the posterior pole.

Confocal near infrared reflectance scanning laser ophthalmoscopy (SLO) utilizes long wavelengths of approximately 820 nm and also captures an en face view of the superficial retinal vessels.^1^ SLO images are typically captured simultaneous to optical coherence tomography (OCT) scans of the inner retinal layers (Supplementary Fig. S1) acting as a localizer to position the OCT beam at the back of the eye accurately and typically images a restricted field of view of 30 degrees. Additionally, confocal imaging leverages laser-point raster scanning to prevent stray light interference during acquisition,^2^ thus providing higher contrast visualization of the en face retinal vasculature over regular color fundus images.

The interferometry of OCT imaging allows for micron-level axial depth visualization on the cross-section, which is independent of biometric factors like axial length and corneal or refractive properties of the eye.^3^ When these parameters are accounted for, the transverse plane can also be imaged with micron-level precision, enabling standardized physical measurements of the en face retinal vessels using microns-per-pixel conversion factors.^4^ In contrast, color fundus imaging typically relies on heuristic methods, such as optic disc area normalization, to achieve standardized measurements.^5^ This suggests that SLO imaging could potentially provide more accurate and clinically relevant measurements of en face retinal vessels compared to traditional fundus imaging, given known biometric factors of the eye are accounted for. Nevertheless, despite the high level of detail captured by SLO images, they are often overlooked, as the primary focus of OCT imaging remains on measuring the cross-sectional retinal layers.

This improved visualization, possibly increased accuracy in measurement and growing abundance from OCT capture make the SLO imaging modality an exciting frontier of en face retinal vessel analysis. Manual delineation of en face retinal vessels is prohibitively time-consuming, labor-intensive, and prone to inaccuracies. Consequently, extensive research has focused on segmenting retinal vessels, primarily in the color fundus modality,^6^ with less emphasis on SLO images.

At the time of writing, very few methods have been reported for retinal vessel analysis on SLO images,^7^^–^^11^ and none for other major landmarks on the SLO image, such as the fovea and optic disc. Additionally, none of these methods have been made available to the research community, which has the potential to impact the standardization of SLO-derived retinal vascular metrics.

Equipping automatic imaging methods with the tools for measuring downstream features for research and clinical use is an important element of any research pipeline.^12^^–^^16^ This allows the general researcher to use these methods in an end-to-end manner, and analyze their own retinal images without specialist training or background in image processing. Unfortunately, there is a lack of such pipelines for SLO images.

Accordingly, we aimed to develop and release a fully automatic analysis toolkit, SLOctolyzer, for segmentation and measurement of retinal vessels as seen on SLO images. Here, we introduce and quantitatively validate the segmentation models, trained on datasets primarily related to systemic health, to detect the en face retinal vessels into arteries and veins, and detect the optic disc and foveal pit.

We highlight several contributions that SLOctolyzer provides to the research community:

• SLOctolyzer is the first open-source pipeline for converting raw SLO images into clinically meaningful, retinal vascular parameters.
• SLOctolyzer's measurement module computes several summary measures of the retinal vessels, such as fractal dimension, vessel density, tortuosity density, vessel caliber, and central retinal artery/vein equivalents.
• SLOctolyzer works on macula- or optic disc-centered images which usually accompany OCT capture.
• SLOctolyzer supports manual annotation to correct erroneous retinal vessel segmentation maps.
• SLOctolyzer is suitable for batch processing large amounts of SLO images, collating results for ease of segmentation quality inspection, and downstream statistical analysis.

## Methods

**Figure 1.**
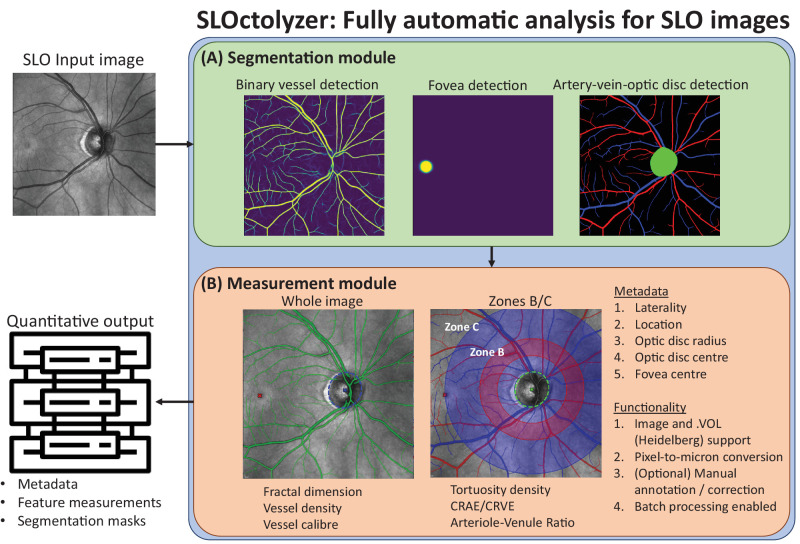
Summary of SLOctolyzer's analysis pipeline with a segmentation module (**A**) for binary vessel/artery/vein segmentation and fovea/optic disc detection, and a measurement module (**B**) for measuring retinal vascular parameters, with additional functionality for metadata extraction and manual annotation.

Figure 1 summarizes the key elements of SLOctolyzer's pipeline, which contains a segmentation module and a measurement module (of which the latter is based on features and code produced by Automorph^13^). We have released SLOctolyzer in a frictionless manner so that it can be used without author permissions, proprietary software, or specialist training. SLOctolyzer is freely available here at https://github.com/jaburke166/SLOctolyzer.

### Data

Data used to build the segmentation module include RAVIR,^11^ a publicly available dataset of pathological SLO images with the arteries and veins segmented, one cohort of healthy volunteers and three cohorts related to systemic health: i-Test,^17^ a cohort of pregnant women at late gestation who are experiencing either a normative, pre-eclamptic, or fetal growth restricted pregnancy; PREVENT,^18^^,^^19^ a cohort of mid-life individuals, half of whom are at risk of developing later life dementia; FutureMS,^20^^,^^21^ a cohort of individuals with newly diagnosed relapsing-remitting multiple sclerosis (MS). All studies and cohorts adhered to the Declaration of Helsinki and received relevant ethical approval, and informed consent from all subjects was obtained in all cases from the host institution.

Table 1 summarizes image characteristics of each cohort. All SLO images were captured using a spectral domain Heidelberg Spectralis HRA + OCT imaging device (Heidelberg Engineering, Heidelberg, Germany) and were either macula-centered or optic disc-centered. SLO images from every study were grayscale and captured a field of view of 30 degrees at the posterior pole which approximately images a 9 mm^2^ region of interest, using a broadband laser light source with a wavelength of 820 nm. All cohorts used an HRA + OCT Spectralis Standard Module except for the i-Test study, which used a portable version, the Spectralis Flex, for 24 participants out of the 93 available. Given a 30-degree field of view covering approximately a 9 mm^2^ region of interest, the SLO images of these cohorts had either approximately 25 or 50 pixels per degree of visual angle, constituting a total imaging resolution of 768 × 768 or 1536 × 1536 (pixel height × width), which had transversal spatial sampling length scales of approximately 11.71 microns-per-pixel or 5.85 microns-per-pixel, respectively.

**Table 1.** Image Characteristics of the Five Cohorts Used to Build SLOctolyzer's Segmentation Module

Study	Participants	Images	Right Eyes	Retinal Pathology	HRA + OCT Module	Image Resolution, px	Location
RAVIR [11]	23	23	14	Yes	Standard	768 × 768	Disc
i-Test [17]	93	186	93	No	Standard and FLEX	768 × 768	Macula
PREVENT [18, 19]	144	285	142	No	Standard	1536 × 1536	Disc
FutureMS [20, 21]	15	15	9	No	Standard	1536 × 1536	Disc
Healthy [22]	7	7	7	No	Standard	1536 × 1536	Disc

HRA, Heidelberg retina angiography; OCT, optical coherence tomography; px, pixels.

Image resolution is in pixels (for both lateral and axial directions), location refers to the centering of the scan, that is, if its macula-centered or disc-centered.

SLOctolyzer's segmentation module contains three segmentation models, one for binary vessel detection, one for fovea detection, and another for artery vein and optic disc detection. Table 2 outlines which cohorts and how many eyes/images of the cohorts each model used for training and evaluation. For all studies except for FutureMS, we selected all available participants for modeling. For FutureMS, a subset of 15 participants from the wider cohort^21^ were selected. These images were chosen based on image features relevant for vessel segmentation, such as blur, illumination, contrast, and abnormal features like vessel tortuosity or optic nerve head atrophy (Supplementary Fig. S2). Similarly, for the i-Test cohort, 15 participants were selected from the wider cohort for building the artery vein optic disc (AVOD) model.

**Table 2.** Number of Eyes (Images) Used for Each Segmentation Model Stratified by Cohort

	Cohort (Eyes/Images)					
Model	RAVIR [11]	Healthy [22]	PREVENT [18, 19]	i-Test [17]	FutureMS [21, 22]	Total
Vessel	23	7	X	X	X	30
Fovea	23	7	285	186	15	516
AVOD	X	X	X	15	15	30

AVOD, artery-vein-optic disc.

### Ground Truth Labels

#### Binary Vessel Detection

A total of 30 SLO images (RAVIR & Healthy; see Table 2) were used to train and evaluate the segmentation model for binary vessel detection. Twenty-three of these were sourced from the RAVIR dataset, of which manual pixel-level annotation of the arteries and veins were done by the same experienced retinal image grader.^11^ Segmentation over the optic nerve head was only performed if the artery and vein classes were able to be resolved. Vessel labels were defined as both the artery and vein class combined. The remaining seven SLO images were made available from healthy eyes and were manually segmented by an experienced image analyst (author S.G.) using ITK-Snap,^23^ which supports pixel-level annotation.

#### Fovea Detection

Fovea detection was performed by the same experienced grader (author J.B.) and used all 516 SLO images.

The definitions of the fovea are guided by features on the image, such as brightness and shape, rather than being biologically driven. We define the foveal pit as a single pixel coordinate on the en face SLO and cross-sectional OCT B-scan at the center of the foveola centralis. The foveola is a circular zone of approximately 350-micron width which is avascular, creating a depression at the center of the macula which pushes the inner retinal layers laterally.^24^ On an OCT B-scan, the depression appears as a dip in the retina and is identified as a pixel at the center of this dip, often aligning with a ridge formed at the photoreceptor layer. On the en face SLO, the depression appears as a dark spot (sometimes showing a hyper-reflective dot in its center) where smaller arterioles and venules, branching from major arteries and veins, converge as they thin in caliber in the macula. See Supplementary Figure S3 and Figure S4 and supporting text for exemplar en face and cross-sectional images with arrows indicating the detected foveal pit.

For the i-Test cohort, access to both the SLO and OCT was possible, which allowed the fovea coordinate on the SLO to be cross-referenced using its transversal position on the corresponding fovea-centered OCT B-scan. The fovea location on each OCT B-scan was detected using Choroidalyzer,^16^ a fully automatic toolkit for choroid analysis and fovea detection in OCT images. For the remaining images, only the SLO was available and the fovea coordinate was selected manually, using an in-house GUI written in Python (version 3.11.6).

We assessed the accuracy of single-grader fovea detection through a grader (author J.B.) re-selecting the fovea pixel coordinate for all 516 images 2 months after the initial manual grading, the results of which can be found in Supplementary Table S1. Repeatability against the initial i-Test fovea coordinates acted as comparison to the ground truth, and the remaining images were used to assess intra-rater repeatability. Intra-rater repeatability had an average intra-class correlation ICC(3, 1) of 0.99 for both *x* and *y* coordinates, and comparison to the ground truth scored an average ICC of 0.82 across both *x* and *y* coordinates.

The final fovea coordinate used for modelling was computed as the average of the two pixel coordinates selected.

#### Artery Vein Optic Disc Detection

Three experienced image analysts (authors J.B., S.G., and A.T.) were each given 14 SLO images to manually annotate the arteries, veins, and optic disc. For each SLO image, a binary vessel map was generated using the binary vessel detection model. Each grader then used ITK-Snap^23^ for anatomic segmentation of arteries, veins, and the optic disc. See Supplementary Figures S5 and S6 for the vessel labeling rules and segmentation protocol each rater followed for consistent and comparative segmentation.

Each set of 14 images had 2 images with distinct overlap between pairwise graders. Thus, there were a total of 30 unique SLO images used for modeling: 15 from the i-Test cohort and 15 from the FutureMS cohort. This overlap allowed for a total of six SLO images which could be used for inter-rater assessment. We calculated inter-rater agreement for all-vessel, artery, vein, and optic disc segmentation using the Dice coefficient, whose results can be found in Supplementary Table S2. All values were found to be greater than 0.93, suggesting an excellent degree of consistency between graders.

We also had a clinical ophthalmologist (author I.M.) qualitatively rate the artery and vein segmentations for all 30 SLO images. For each SLO image, we asked the ophthalmologist (author I.M.) to rate its image quality and the segmentation quality of the artery and vein annotation using a 5-point ordinal scale between −2 (very bad) and 2 (very good). The results of this rating can be found in Supplementary Table S3. Sixty-three percent (19/30) were rated as “very good,” with the remaining annotations graded as “good,” except for only one which was rated as “okay.” This example can be seen in Supplementary Figure S7, which had 2, small miss-labeled vessels. Importantly, there were no “bad” or “very bad” ratings for these manual annotations.

#### The RAVIR Dataset

The RAVIR dataset^11^ was intended to be used for training the AVOD model, as it contains suitable examples of severe retinal pathology. However, before modeling we reviewed the segmentation labels and observed inconsistent artery and vein classification, an example of which can be seen in Supplementary Figure S8. Therefore, we decided to use the RAVIR dataset as an external test set for the AVOD model, after correcting for any misclassified veins or arteries manually using ITK-Snap.^23^ This external test set presents a significant challenge to the AVOD model as it contains examples of severe retinal pathology.

Before evaluating the AVOD model, the corrections to the RAVIR dataset were reviewed by a clinical ophthalmologist (author I.M.) against the original labels in a masked, randomized fashion. For each image, the ophthalmologist was presented with two segmentation labels without revealing their source. He was then asked to rate the SLO image quality and each artery-vein classification using a 5-point ordinal scale from −2 (very bad) to 2 (very good), and select which of the labels he preferred, with options for none or both. The results are shown in Supplementary Table S4, and the ophthalmologist preferred over 64% (14/23) of the corrected labels, and the labels for the remaining images were equally of good quality, according to the ophthalmologist. Additionally, we segmented the optic disc for this external test set for comparison with our optic disc segmentation.

### Segmentation Module

During training, we applied the same data augmentations randomly to each model's training data. This included random horizontal and vertical flips (*P* = 0.5), affine transformations to allow random rotation and scaling, uniformly sampled in [0, 90] and [0.4, 1.6], respectively (*P* = 1/3). We also applied a Gaussian blur with random window size and standard deviation (SD) uniformly sampled in [1, 20] and [0.1, 4.0], respectively (*P* = 1/4). Finally, we applied random brightness and contrast adjustments, altering the brightness and contrast using uniformly sampled scale factors [0.8, 1.2] and [0.75, 1.25], respectively.

We used python 3.12, PyTorch 2.0, Segmentation Models PyTorch^25^ and MONAI.^26^

#### Binary Vessel Detection

The model and training pipeline has been discussed previously.^27^ Briefly, the model was a custom UNet deep learning architecture trained from scratch, with details on how to reproduce the model architecture shown in Supplementary Figure S9 with surrounding text.

The model was trained on a total of 30 SLO images. The dataset was split randomly into training (*n* = 24), validation (*n* = 2), and test (*n* = 4) sets. Offline data augmentation randomly cropped each image into 20 patches of size 320 × 240 to artificially increase the training set, reduce computational load, and accelerate the training rate.

The model was trained to minimize the Dice Focal loss,^26^ using the Adam^28^ optimizer (with default optimizer hyperparameters) for 600 epochs using a batch size of 20. The initial learning rate was set as 1e-3 until epoch 300, at which point the learning rate was reduced to 1e-4 for the remainder of training.

#### Fovea Detection

Fovea segmentation is a significantly easier task than vessel detection or artery-vein classification, so our model used a pretrained,^25^ lightweight UNet deep learning architecture with a MobileNetV3^29^ backbone, previously trained using ImageNet.^30^ Details on how to reproduce the model architecture are shown in Supplementary Code Listing 1 with surrounding text.

This model used all available images from all cohorts, comprising 516 SLO images. The dataset was split into training (*n* = 359), validation (*n* = 104), and test sets (*n* = 54) at the participant level, so there was no overlap of participants between sets. All images were resized to a common image resolution of 768 × 768 pixels for training. To facilitate learning through segmentation, training labels were circular binary masks whose center was the fovea coordinate with a radius of 60 pixels.

The model was trained to minimize the binary cross entropy loss using the AdamW^31^ optimizer for 200 epochs using a batch size of 8. During training, we applied two sets of random augmentations per image, per epoch, artificially doubling the batch size. An initial learning rate of 5e-4 was set, and after a 10-epoch linear warm up to a learning rate of 5e-3, it decayed following a cosine relationship for 90 epochs, with the learning rate set at 5e-4 for the remaining 100 epochs.

#### Artery Vein Optic Disc Detection

Given the number of segmentation tasks and dataset size, we similarly opted for fine-tuning a pretrained UNet deep learning model, this time using a deep ResNet101^32^ backbone, previously trained on ImageNet.^30^ Details on how to reproduce the model architecture are shown in Supplementary Code Listing 1 and surrounding text.

The AVOD model was trained on a total of 30 SLO images. The dataset was split into training (*n* = 20), validation (*n* = 4), and test (*n* = 6) sets, with equal proportion of cohorts (and thus macula and disc-centered images) in each set. All images were resized to a common image resolution of 768 × 768 pixels for training.

The model was trained to minimize the Dice Focal loss,^26^ using the Adam^28^ optimizer (with default optimizer hyperparameters) for 300 epochs using a batch size of 4, and a learning rate of 5e-4.

### Measurement Module

**Figure 2.**
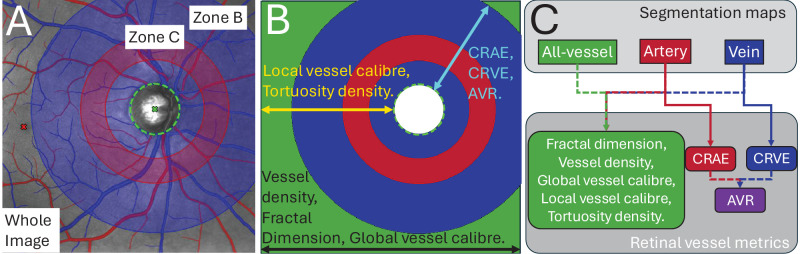
(**A**) Disc-centered SLO image with segmentations and regions of interest overlaid. (**B**) Region of interest masks for the whole image (*green*), zone C (*blue*), and zone B (*red*) with the names of vessel metric, color coordinated with *arrows* indicating which regions they are measured in. (**C**) Flowchart of which vessel metrics are computed for which segmentation map.

SLOctolyzer's second module is a suite of tools for measuring retinal vascular parameters and are based on the features measured when processing color fundus images using Automorph.^13^ Figure 2 shows the different regions of interest defined and the measurements taken per region and per vessel map.

We define only one region of interest for macula-centered images and three regions of interest for disc-centered images to compute feature measurements on. The region of interest common across the macula- and disc-centered images is the whole image (see Fig. 2B, green). Relevant to only disc-centered images are the second and third regions of interest, which define two concentric rings centered at the optic disc called zones B and C.^22^ Zone B takes an annulus from one-half diameter (D) away from the optic disc margin to a full diameter away (0.5 D − 1 D; see Fig. 2B, red), and zone C measures from the optic disc margin to two diameters away (0 D – 2 D; see Fig. 2B, blue).

Figure 2B shows which metrics are measured across which regions of interest. Across the whole image, we measured the global features of density, complexity, and caliber, computed using vessel density (the ratio of vessel pixels to all pixels), fractal dimension (Minkowski-Bouligand dimension)^33^ and global vessel caliber (ratio of vessel pixels to skeletonized vessel pixels) — these measurements are not taken for localized zones B and C. For every region of interest (whole image and zones B and C), we measured local vessel caliber and tortuosity density^34^ across individual vessel segments whose end points are defined by arteriovenous crossing and bifurcations. Global and local vessel caliber are very similar metrics, but the latter provides a more granular approach to computing caliber from individual vessel segments — see Supplementary Figure S10 and supporting text.

Figure 2C shows a simple flowchart of which measurements are taken across which vessel map. All of these aforementioned features are measured across artery, vein and all-vessel maps (see Fig. 2C, green). For artery and vein vessel maps only, we measured central retinal artery and vein equivalents (CRAE and CRVE) using the Knudston approach,^35^ respectively, as well as the arteriole-to-venule (AVR) ratio (see Fig. 2C, red, blue, and purple). Note that although the CRAE/CRVE/AVR are typically only measured for zones B and C in disc-centered images, they are also computed across the whole image and for macula-centered images too.

### SLOctolyzer's Pipeline

Figure 1 describes the analysis pipeline for SLOctolyzer. Please see Supplementary Figures S11 and S12 with supporting text for more information on SLOctolyzer's setup, interface, and output.

#### Input

SLOctolyzer supports macula-centered and disc-centered SLO images inputted as regular image files (.jpeg, .png, etc.), as well as .vol file formats (from Heidelberg Engineering imaging devices). In the former, the end-user can optionally input information on spatial sampling, laterality (right/left), and location (macula-/disc-centered). If the laterality and location of the SLO image are not inputted, these are inferred based on the detected locations of the fovea and optic disc after segmentation, and measurements are outputted in pixel space. For inputting .vol files, these store the image data and metadata necessary for converting measurements from pixel space to physical space — other file formats such as .e2e and .dcm are currently not supported, but we are working on this presently. A configuration file is also provided for the end-user to specify input/output directories, whether the pipeline skips over files which have unexpected errors during processing (helpful for large-scale batch processing), and the option to ignore saving out segmentation maps (to reduce memory consumption during processing).

#### Segmentation and Measurement

During segmentation, the image is first resized to a common image resolution of 768 × 768 pixels, as the segmentation module was trained on this image resolution, thus making the pipeline invariant to different spatial sampling length scales (assuming a fixed field of view). Moreover, this resizing prevents any segmentation error in images with larger image resolution due to the central artery/vein light reflex^27^ which appears as a bright strip in the center of a vessel, potentially leading to a large vessel being perceived as two smaller and parallel vessels. Upon resizing, full segmentation of the vasculature, optic disc, and fovea is performed and the probability segmentation maps are then resized to the image's original image resolution. After thresholding with a value of 0.5 to produce binary masks, post-processing is applied to remove small false positive regions, and disconnected vessels are joined to their neighboring vessels to remove small gaps.

The foveal pit is defined as the centroid of the fovea binary mask. If the SLO image is disc-centered, the optic disc binary mask is modeled as an ellipse, and its center and diameter are the ellipse's center and the average of its major and minor axis lengths, respectively. If the SLO image is macula-centered, measurements on the all-vessel/artery/vein maps are only taken across the whole image. If the SLO image is disc-centered, measurements are taken across the whole image and for zones B and C, which are defined using the optic disc diameter.

#### Output

Measurements will be saved out per file, alongside a process log and key metadata located in the file's metadata or inferred during analysis (laterality, location, fovea center, optic disc diameter and center). Optionally, segmentation masks will be saved out, as well as helpful visualizations to inspect segmentation quality. If running on a batch of images, a collated .xlsx file will be saved out row-wise compiling all measurements and metadata from all image files analyzed. Finally, a folder of visualizations, superimposing the segmentations onto the SLO image will be saved out per file which will aid segmentation quality checking.

Although we do not have an automatic way to assess segmentation quality, an additional feature of SLOctolyzer is its ability to correct manual segmentations. Given a saved-out segmentation mask of the arteries, veins, and optic disc, the end-user may correct any erroneous areas using ITK-Snap^23^ and save the corrected version in the same folder as the original mask. Upon rerunning the pipeline again, it will use this corrected annotation and re-compute the measurements for that image.

### Statistical Analysis

Each segmentation model, after model selection based on the performance against their respective validation set, was evaluated on their respective, internal test set. Additionally, for the AVOD model, we used the RAVIR dataset^11^ as an external test to measure the segmentation module's robustness to severe retinal pathology.

We used standard segmentation metrics, such as area under the receiver operating characteristic curve (AUC) and the dice similarity coefficient (at a standard threshold of 0.5) to evaluate segmentation performance. We also used mean absolute error (MAE) to measure performance on downstream, retinal vascular parameters fractal dimension, and local vessel caliber (in microns [µm], where transversal spatial sampling length-scales were available and pixels [px] otherwise). We used MAE to measure the error in predicted fovea pixel coordinate (along both axes) and optic disc area.

For internal test set evaluation, the whole image was used as the region of interest due to the low sample size of disc-centered images in each internal test set. For external test set evaluation, all regions of interest (whole image, and zone B and zone C) were evaluated, as RAVIR contains only disc-centered images.

We also measured the reproducibility of SLOctolyzer on downstream retinal vascular parameters, using the first 60 participants (120 eyes) of the i-Test cohort (whose data was available at the time of analysis). SLO images were captured simultaneous to macula-centered, OCT volume capture when enhanced depth imaging mode was toggled on and off, creating a repeated, albeit unregistered, pair of SLO images per eye. We measured all retinal vascular parameters previously mentioned across the whole image, and report mean absolute error and Pearson (P), Spearman (S), and intra-class correlation ICC(3, 1) coefficients for population-based reproducibility analysis. We also report Bland-Altman plots^36^ to assess the distribution of residuals.

Additionally, we report the reproducibility of SLOctolyzer at the eye-level using a measure of individual-level measurement noise λ.^37^ We express the variability of each measured feature within an eye in units of the feature's overall population variability. Measurement noise λ is computed per feature, per eye, as the ratio between the standard deviation of within-eye measurements and the standard deviation of between-eye measurements, across the population. For convenience, *λ* is presented as a percentage, with 0 as the optimal value. Note that the population are all White women with a mean (SD) age of 34.7 (5.2) years, at mean (SD) gestation of 35.6 (3.4) weeks, and thus we do not expect there to be huge variation across the population.

Finally, we measured the execution time of SLOctolyzer's segmentation module and the whole pipeline on macula- and disc-centered images of different image resolutions. We ran each experiment 100 times and report the mean and SD execution time in seconds. Timed experiments were run on a Windows laptop with a 4-year-old Intel Core i5 (8th generation) CPU and 16 Gb of RAM. For brevity, we will refer to this as the “laptop CPU” in the rest of the text.

## Results

### Evaluation

**Figure 3.**
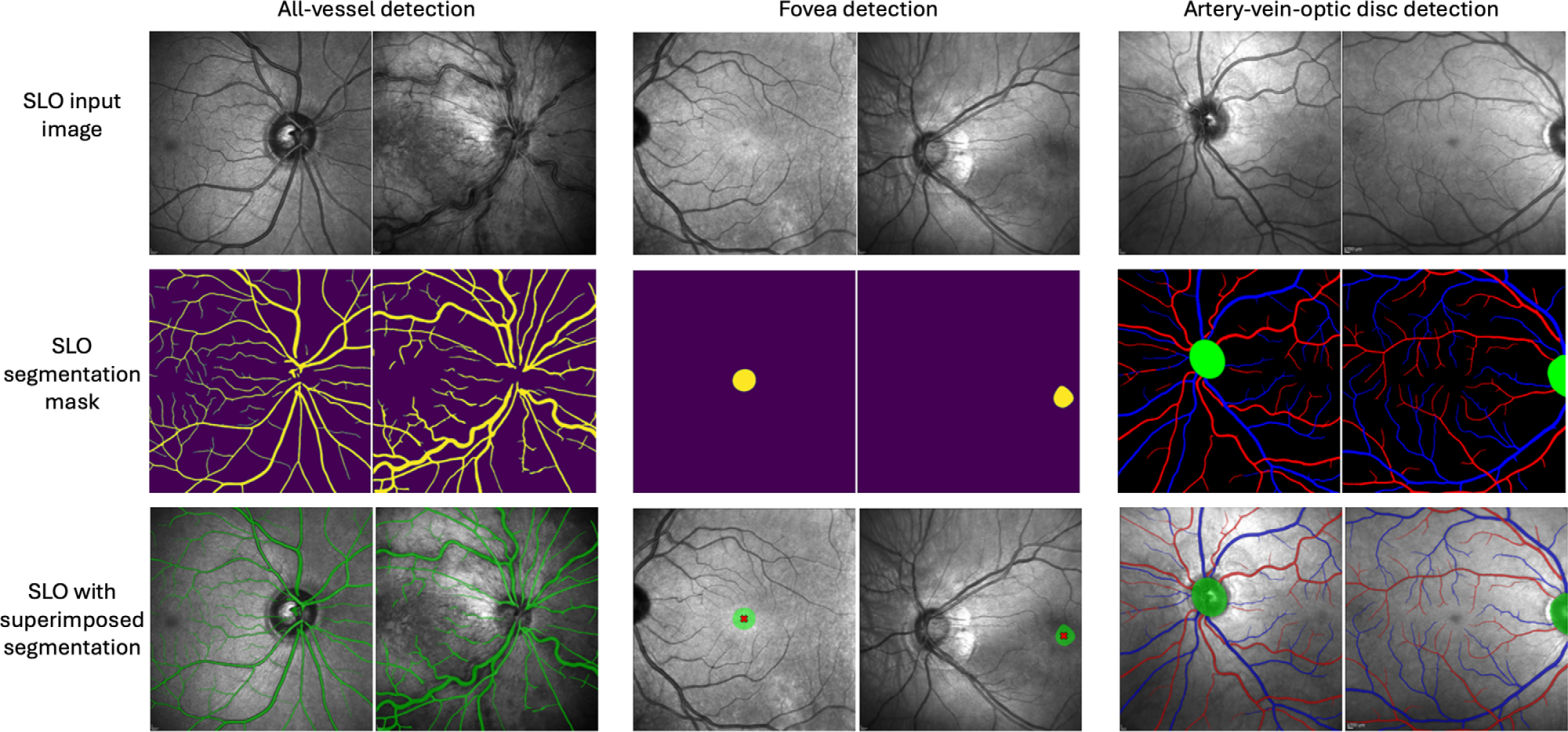
Predicted segmentation masks from each model for two examples randomly selected from their internal test sets.

Segmentation results are presented in Table 3 for all 3 models for their respective internal test sets. All models had a strong AUC score (all greater than 0.98), suggesting the raw probability maps were well calibrated in being able to score highly the relevant pixels related to each of their segmentation tasks, with the fovea and optic disc detection performing best, which was to be expected because these are easier segmentation tasks than vessel detection. Upon thresholding, both binary vessel and AVOD models scored high dice scores for all-vessel detection (binary vessel model = 0.91 and AVOD model = 0.86), and the AVOD model scored slightly lower dice scores for artery and vein classification (artery = 0.84 and vein = 0.85). This was unsurprising, because distinguishing between an artery and a vein is significantly more challenging than differentiating retinal vasculature from retinal tissue. Figure 3 shows example segmentation outputs from the internal test sets.

**Table 3.** Segmentation Results for Each Segmentation Task (Top). Mean Absolute Error Across Relevant Measurements for Each Segmentation Task (Bottom)

	Vessel		Fovea		Artery		Vein		Optic Disc	
Metric	AUC	Dice	AUC	Dice	AUC	Dice	AUC	Dice	AUC	Dice
Segmentation	0.99	0.91	0.99	0.88	0.99	0.84	0.99	0.85	0.99	0.94
	FD	Caliber [px]	C_*x*_ [px]	C_*y*_ [px]	FD	Caliber [px]	FD	Caliber [px]	Area [px^2^]	

Feature	0.01	0.15	6.28	4.78	0.02	0.21	0.01	0.15	638.17	

Caliber, local vessel caliber; FD, fractal dimension; C*_x_*/C*_y_*, mean absolute error in the horizontal/vertical component of the centroid for the fovea; px, pixels.

The MAE for fovea coordinate detection was 6.3 and 4.8 pixels for the horizontal and vertical axes (see Table 3), thus reporting an MAE of 7.9 pixels diagonally. Images had an image resolution of 768 × 768 pixels, covering approximately a 9 mm^2^ region of interest, which makes their transversal spatial length-scale approximately 11.71 microns-per-pixel in both axes. Thus, this diagonal MAE corresponds to approximately 92.7 microns, which is well within the estimated 350-micron width of the foveola centralis.^24^

Similarly, an MAE of 638 square pixels for the optic disc area corresponds to approximately an MAE of 0.08 mm^2^, which is not clinically significant given the wide distribution of optic disc sizes,^38^ the observed change across the myopic spectrum^39^ and even differences across different devices.^40^ MAE for local vessel caliber was around 0.2 pixels, which corresponds to approximately 2.32 microns, and is similarly an insignificant error given population distributions for arterioles and venules using confocal SLO imaging.^41^

**Figure 4.**
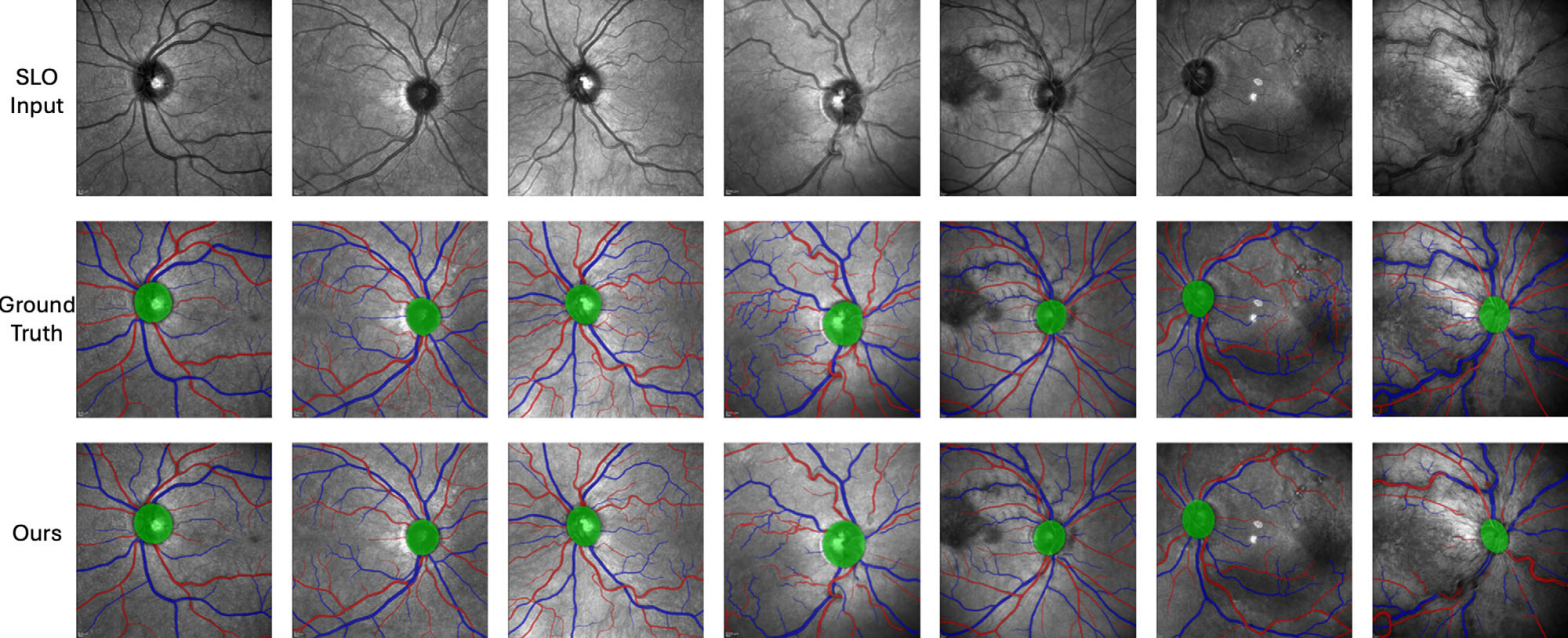
Visual comparison between ground truth and predicted segmentations for the external test set, RAVIR, for artery-vein optic disc detection. The images were selected to show progressing eye pathology from left to right.

The results of our segmentation module evaluated against the external test set is shown in Table 4. The RAVIR dataset^11^ presents a significant challenge due to retinal pathology heavily featuring in the 23 SLO images. The model performs worse compared with the unseen, internal test data which relate to systemic health (dice coefficient averaged across zones: artery = 0.72; vein = 0.76; and optic disc = 0.90), but quantitatively performs well for those images with mild pathology. Given that the segmentation models were trained on images with grossly normal retinae related to systemic disease, it is perhaps no surprise that the performance of the model decreases as the extent of retinal pathology increases. Figure 4 qualitatively compares the ground truth labels (middle row) with SLOctolyzer's predictions (bottom row), for SLO images of increasing retinal pathology from left-to-right. Whereas the major vessels are classified correctly for the majority of cases, as the pathology becomes more severe, we see smaller and more tortuous vessels ignored, and segmentations of the major vessels misclassified.

**Table 4.** Segmentation and Feature Measurement Mean Absolute Errors of the Artery Vein Optic Disc (AVOD) Segmentation Model Against the RAVIR External Test Set

	Artery				Vein				Optic Disc		
Region of Interest	AUC	Dice	FD	Caliber [px]	AUC	Dice	FD	Caliber [px]	AUC	Dice	Area [px^2^]
Whole Image	0.94	0.72	0.04	0.69	0.96	0.75	0.07	0.98	0.97	0.9	788.5
Zone B	0.99	0.73	0.04	1.01	0.99	0.77	0.06	1.18			
Zone C	0.96	0.72	0.04	0.79	0.97	0.76	0.05	1.15			

Caliber, local vessel caliber; AUC, area under the receiver operating characteristic curve; FD, fractal dimension; px, pixels.

### Reproducibility

Table 5 presents correlation values for all retinal vascular parameters for the binary vessel segmentation model (all-vessel), and the AVOD model. All correlations showed strong or excellent agreement among features, with fractal dimension and vessel density scoring the highest. Across the cohort, veins appeared as the poorest performing, but not by any significant margin.

**Table 5.** Population-Based Reproducibility Performance of SLOctolyzer, Reporting Mean Absolute Error (MAE), Pearson (P), Spearman (S), and Intra-Class Correlations ICC(3, 1)

	All-Vessel			Artery			Vein		
Metric	MAE	P/S	ICC (3, 1)	MAE	P/S	ICC (3, 1)	MAE	P/S	ICC (3, 1)
Fractal dimension	0.008	0.93/0.92	0.97 (0.95, 0.98)	0.005	0.95/0.94	0.97 (0.96, 0.98)	0.007	0.89/0.89	0.94 (0.92, 0.96)
Vessel density	0.001	0.95/0.95	0.97 (0.96, 0.98)	0.002	0.94/0.94	0.97 (0.95, 0.98)	0.001	0.94/0.92	0.97 (0.95, 0.98)
Global caliber [µm]	1.58	0.88/0.89	0.93 (0.90, 0.95)	1.6	0.84/0.83	0.91 (0.87, 0.94)	1.61	0.87/0.87	0.93 (0.90, 0.95)
Local caliber [µm]	1.5	0.87/0.88	0.93 (0.89, 0.95)	1.47	0.85/0.85	0.92 (0.88, 0.94)	1.41	0.87/0.87	0.93 (0.90, 0.95)
Tortuosity density	0.03	0.74/0.75	0.85 (0.79, 0.9)	0.01	0.80/0.80	0.89 (0.84, 0.92)	0.02	0.70/0.68	0.83 (0.75, 0.88)
AVR/CRAE/CRVE [µm]	0.03	0.77/0.79	0.87 (0.82, 0.91)	6.28	0.85/0.86	0.92 (0.88, 0.94)	10.1	0.79/0.80	0.89 (0.83, 0.92)

All Pearson and Spearman correlations were statistically significant with *P* values *P <* 0.0001. For readability, AVR/CRAE/CRVE were combined into one row — AVR is dimensionless and specified in the all-vessel column, whereas CRAE and CRVE are in microns and only report for the artery and vein maps, respectively.

**Figure 5.**
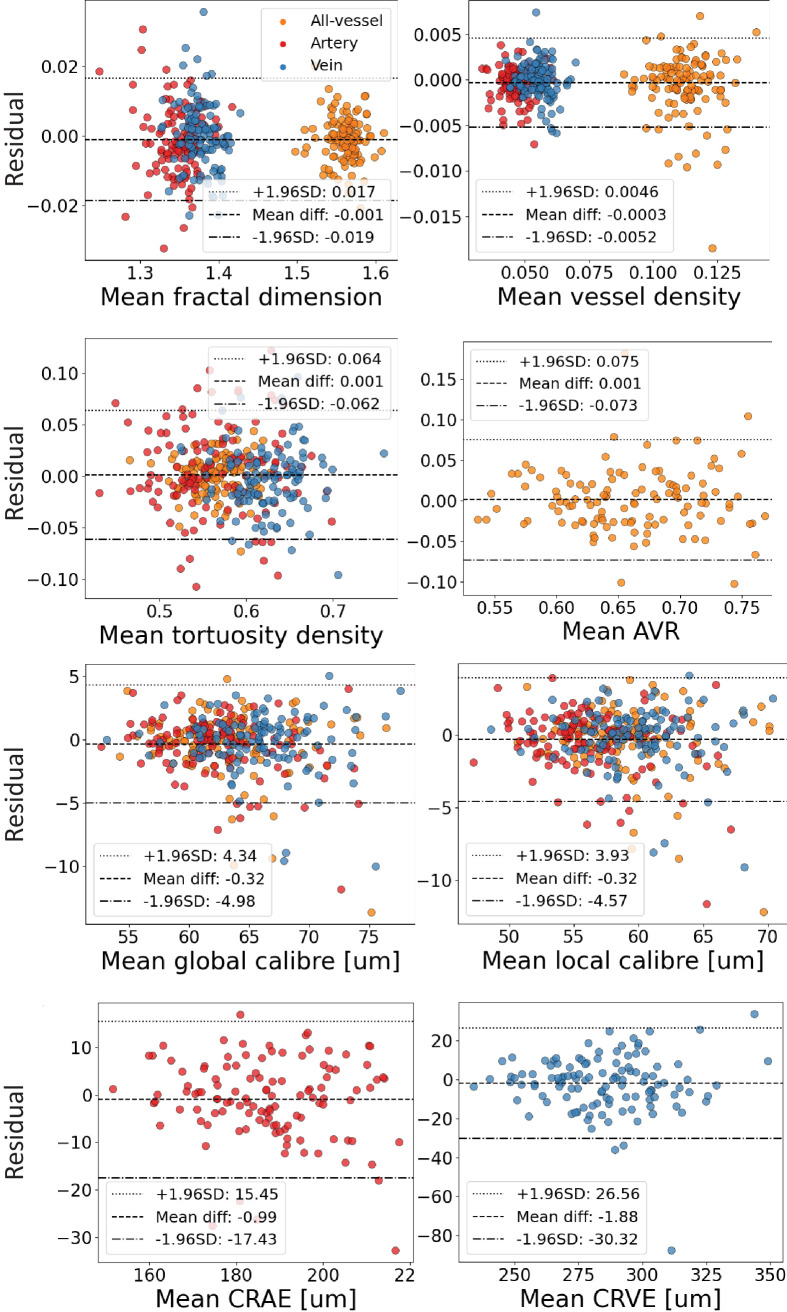
Bland-Altman plots of residual distributions from assessing the reproducibility of SLOctolyzer's segmentation module across all retinal vessel metrics. Scatter-plot residuals for arteries (*red*), veins (*blue*), and all-vessel (*orange*) are overlaid together.

Figure 5 shows Bland-Altman plots of the residuals between paired, repeated SLO images. We found that SLOctolyzer was highly reproducible, with the distribution of residuals according to most metrics centered around 0, showing no apparent trend. For fractal dimension, there was a mean difference (MD) of −0.001 with limits of agreement (LoA) of [−0.019, 0.017]; for vessel density there was an MD of −0.0003 and LoA of [−0.0052, 0.0046]; for global vessel caliber there was an MD of −0.32 µm and LoA of [−4.98, 4.34]; for local vessel caliber there was an MD of −0.32 µm and LoA of [−4.57, 3.93]; for tortuosity density there was an MD of 0.001 and LoA of [−0.062, 0.064]; for CRAE there was an MD of −0.99 µm and LoA of [−17.43, 15.45]; for CRVE there was an MD of −1.88 µm and LoA of [−30.32, 26.56]; finally, for AVR there was an MD of 0.001 and LoA of [−0.073, 0.075].

**Figure 6.**
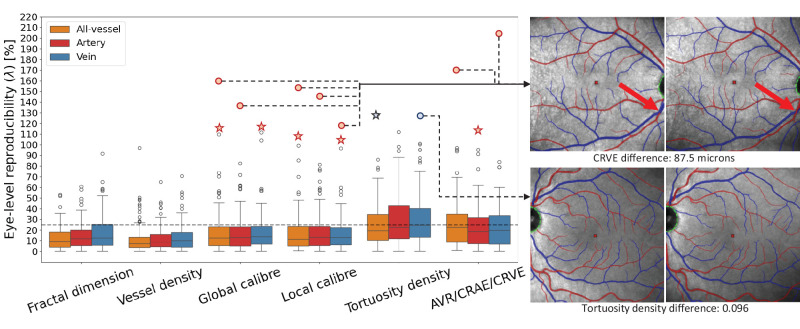
Reproducibility at the eye-level of each of the features outputted by SLOctolyzer. Box-plot distributions are shown for each feature, with all-vessels in orange, arteries in *red* and veins in *blue*. Note that the AVR/CRAE/CRVE features are presented together as CRVE/CRAE are measured across arteries (*red*) and veins (*blue*) separately, whereas AVR is a combination of the two, thus represented in the all-vessel class (*orange*). A horizontal, *dashed line* at the *λ* = 25% mark is shown as a visual aid. We also show the eyes (with segmentations overlaid) which had the greatest eye-level reproducibility measurement error for large-vessel caliber and vein tortuosity density (circular scatter-points). Eyes corresponding to the star-shaped scatter-points are shown in Supplementary Figure S13.

Figure 6 reports the eye-level reproducibility of each of the features measured by SLOctolyzer. For fractal dimension, vessel density, and global/local caliber, the upper, or third quartile is below 25% across all vessel types. Thus, the range of measurement error for these features in the majority of the paired, but unregistered, eyes were below 25% of the overall populations’ variability. Given the homogeneous nature of the population's demographics, this is very reasonable. Moreover, vessel density has the smallest interquartile range among features (mean all-vessels, *λ* = 11.7%; arteries, *λ* = 12.1%; and veins, *λ* = 13.3%), suggesting this feature, closely followed by fractal dimension, had the least measurement noise. Tortuosity density and AVR/CRAE/CRVE appear as the most sensitive features, with veins as the most variable relative to arteries and all-vessels.

The eyes with the worst reproducibility for vessel caliber and tortuosity density are shown to the right of the box plots, with dashed lines linking the major outlier scatter-points to the corresponding eye's SLO image. For all caliber metrics, the worst metric came from the same eye. This was due to an arteriovenous crossing near the edge of the image, where a major artery and vessel overlapped, and SLOctolyzer classified the artery and vein differently (red arrows). This led to a large absolute difference of 87.5 microns in CRVE. For tortuosity density, the vessel maps do not look qualitatively different, but the absolute difference was 0.096. We believe that this is due to the tortuosity density's sensitivity to arteriovenous crossings which disconnect the respective artery and vein vessel maps. Star-shaped scatter-points represent second to largest outliers for vessel caliber and tortuosity density and are shown in Supplementary Figure S13, and are similarly due to arteriovenous crossings or vessel disconnectedness.

### Execution Time

Table 6 shows the execution time of SLOctolyzer's segmentation module and pipeline. Because the segmentation module resizes all images to a common image resolution of 768 × 768 pixels, the segmentation execution time across different locations and image resolutions are approximately equal. For binary vessel segmentation, it takes approximately 4.5 seconds per image, for the fovea detection model it takes approximately 0.6 seconds per image, and, for AVOD detection, it takes approximately 3.0 seconds per image. Thus, SLOctolyzer's segmentation module using the laptop CPU takes approximately 11.1 ± 0.5 seconds per image. Measuring the execution time of the entire pipeline on the laptop CPU took 18.9 ± 1.0 seconds for a macula-centered SLO with image resolution 768 × 768 pixels, 28.6 ± 1.0 seconds for a disc-centered SLO with image resolution 768 × 768 pixels, and 111.0 ± 3.5 seconds for a disc-centered SLO with image resolution 1536 × 1536 pixels. Disc-centered SLO images are more time intensive as measurements are made across the whole image, and for zones B and C across the all-vessel, artery and vein segmentation maps separately.

**Table 6.** Average (Standard Deviation) Execution Time in Seconds of the Segmentation Module and SLOctolyzer's Entire Pipeline for SLO Images of Different Location and Image Resolution

	Segmentation Module			
Image Type	Binary Vessel	Fovea	AVOD	Whole Pipeline
Macula-centered (768 × 768)	4.8 (0.407)	0.6 (0.067)	2.9 (0.408)	**18.9 (0.984)**
Disc-centered (768 × 768)	4.3 (0.147)	0.6 (0.047)	2.9 (0.440)	**28.6 (1.040)**
Disc-centered (1536 × 1536)	4.2 (0.191)	0.6 (0.051)	2.7 (0.133)	**111.0 (3.540)**

## Discussion

We have developed a fully automatic method, SLOctolyzer, for processing macula-centered and disc-centered SLO images which typically accompany OCT data but are often discarded in research. SLOctolyzer converts a raw SLO image into reproducible and clinically meaningful retinal vascular parameters, including fractal dimension, tortuosity, and vessel caliber.

SLOctolyzer performed well on unseen data, but we observed a performance drop against an external test set featuring severe retinal pathology. SLOctolyzer's segmentation module was trained on datasets primarily related to systemic health and was thus designed for modeling the retinal vasculature in these disease contexts, so there was understandably a noticeable decrease in performance as the subjective extent of pathology increased. However, we believe that SLOctolyzer's additional functionality to correct any erroneous segmentation maps and re-compute measurements will prove very useful for cases of severe retinal pathology. Regardless, we envision SLOctolyzer being a very useful tool for SLO image analysis in systemic disease, particularly as the nascent field of oculomics increases rapidly^42^ and the populations being studied often have grossly normal retinae.

SLOctolyzer's segmentation module had good reproducibility, with the residuals across all metrics for vessels, arteries, and veins exhibiting no pattern and being centered around 0. In particular, the residuals for measurements of global and local caliber (in microns) appear to show proportionately small error in comparison to what is generally considered as clinically significant. Meta-analyses have found vessel caliber difference of 10 to 15 microns between middle-age and old-age, with a 20 micron increase in venule caliber associated with increased risk of stroke by 1.15.^43^

The distributions of measurement error at the eye-level for fractal dimension, vessel density, and local/global caliber were found to be reasonable given the homogeneous nature of the iTest cohort studied for reproducibility — with tortuosity density and large-vessel caliber having higher variability. We observed that small differences in the segmentation maps can have an inordinate impact on tortuosity density (see Supplementary Fig. S13A). This highlights the apparent sensitivity of tortuosity density, regardless of whether the segmentation looks qualitatively different. Segmentation error does contribute to this error, as arteriovenous crossings result in artery-vein map disconnectedness, which can have an inordinate impact on generating individual vessel segments to measure tortuosity on. This also observation adds to a potential problem surrounding standardization of retinal vascular parameters.^44^

For large-vessel caliber, the largest outliers came from the same eye, which showed a major arteriovenous crossing at the edge of the image causing an artery-vein misclassification. Moreover, large-vessel caliber metrics like AVR/CRAE/CRVE are typically reserved for disc-centered SLO images as these show the major vessels in full as they branch from the optic nerve head. Thus, the unregistered nature of the macula-centered SLO pairs, combined with major vessels potentially cropped toward the edge of the image, likely contributed to the measurement error (see Supplementary Fig. S13B). We hope in the future we may also assess SLOctolyzer's reproducibility for paired, disc-centered SLO images.

On a 4-year-old Intel Core i5 (8th generation) laptop CPU, SLOctolyzer can segment an SLO image in around 11 seconds. For an SLO with image resolution 768 × 768 pixels, the entire pipeline takes 19 seconds for macula-centered images and 29 seconds for disc-centered images. It can be used interactively or for batch processing (see Supplementary Fig. S11), converting raw SLO images into reproducible vascular metrics and outputting segmentation masks for quality inspection (see Supplementary Fig. S12).

There were some limitations associated with this work. First, the SLO images used to build SLOctolyzer's segmentation module were sourced from only one manufacturer (Heidelberg Engineering) and we do not know SLOctolyzer's performance on SLO images from other devices. Moreover, our datasets only contained macula- and disc-centered SLO images captured at a field of view of 30 degrees, and we did not explicitly test the pipeline's performance at different locations or fields of view in the posterior pole.

Furthermore, most of the images were from individuals recruited to studies on systemic disease, which did not have obvious retinal pathology and we therefore expect the segmentation module to not be entirely robust to these cases. Finally, these individuals were of White ethnicity, and so we do not know the pipeline's performance across varying retinal pigmentation. However, SLOctolyzer's additional functionality allows for any unexpected segmentation errors to be corrected manually via ITK-Snap.^23^

A technical limitation of the work is that SLOctolyzer's segmentation module is made up of three distinct models. The rationale for this was clear at the time of model construction: we could leverage an initial binary vessel segmentation model to help us manually annotate the arteries and veins for building an artery-vein detection model, a much more challenging task, with the fovea detection model utilizing all available images due to the speed of ground truth labeling. However, these models now provide a quick and consistent way to annotate large datasets which, after inspection and correction by expert graders, could be used to build a much more robust segmentation model.

Thus, we intend to improve SLOctolyzer's segmentation module in future work by constructing a single model to perform all segmentation tasks, and include a larger and more diverse dataset on which it will be trained and evaluated. These will include examples related to retinal pathology, capturing different regions and fields of view of the retina, and from multiple imaging devices and manufacturers.

We anticipate SLOctolyzer's relevancy for processing 820 nm SLO images which traditionally accompany OCT image capture using Heidelberg Engineering imaging devices. However, further exploration will be conducted on its generalizability to other SLO imaging modalities, such as Optos ultra-wide field SLO,^45^ or Heidelberg's more recent multicolor SLO.^46^

## Conclusions

We have developed a fully automatic method for extracting useful information on the retinal vessels from SLO images, including fractal dimension, tortuosity, and vessel caliber. SLOctolyzer, the first open-source analysis toolkit for macula- and disc-centered SLO images, addresses the lack of open-source tools for this image modality. It can leverage existing OCT datasets to provide reproducible and clinically meaningful retinal vascular parameters from the SLO images which are captured in parallel to OCT acquisition, but are often overlooked. Available on GitHub, SLOctolyzer requires no specialist training or proprietary software and supports batch processing and manual correction. We hope this tool will encourage the research community to reconsider the value of SLO images, facilitate collaboration, and help to standardize retinal vascular parameters in SLO images.

## Supplementary Material

Supplement 1

## References

[1] Terasaki H, Sonoda S, Tomita M, Sakamoto T. Recent advances and clinical application of color scanning laser ophthalmoscope. *J Clin Med*. 2021; 10(4): 718. 33670287 10.3390/jcm10040718 PMC7917686

[2] Hildebrand GD . Chapter 9 - imaging the fundus. In: Hoyt CS, Taylor D, eds. *Pediatric Ophthalmology and Strabismus*. 4th ed. London, UK: W.B. Saunders; 2013: 63–70.

[3] Salmon AE, Sajdak BS, Atry F, Carroll J. Axial scaling is independent of ocular magnification in oct images. *Invest Ophthalmol Vis Sci*. 2018; 59(7): 3037–3040. 30025118 10.1167/iovs.17-23549 PMC6005622

[4] Scoles D, Mahmoud TH. Inaccurate measurements confound the study of myopic macular hole. *Ophthalmol Retina*. 2022; 6(2): 95–96. 35123728 10.1016/j.oret.2021.10.009

[5] Schanner C, Hautala N, Rauscher FG, Falck A. The impact of the image conversion factor and image centration on retinal vessel geometric characteristics. *Front Med*. 2023; 10: 1112652. 10.3389/fmed.2023.1112652 PMC10063888 37007779

[6] Mahapatra S, Agrawal S, Mishro PK, Panda R, Dora L, Pachori RB. A review on retinal blood vessel enhancement and segmentation techniques for color fundus photography. *Crit Rev Biomed Eng*. 2024; 52(1): 41–69. 10.1615/CritRevBiomedEng.2023049348 37938183

[7] Xu J, Ishikawa H, Wollstein G, Schuman JS. Retinal vessel segmentation on SLO image. In: 2008 30th Annual International Conference of the IEEE Engineering in Medicine and Biology Society. *Annu Int Conf* *IEEE* *Eng Med Biol Soc*. 2008; 2008: 2258–2261. 10.1109/IEMBS.2008.4649646 PMC2908151 19163149

[8] Pellegrini E, Robertson G, Trucco E, et al. Blood vessel segmentation and width estimation in ultra-wide field scanning laser ophthalmoscopy. *Biomed Opt Express*. 2014; 5(12): 4329–4337. 25574441 10.1364/BOE.5.004329 PMC4285608

[9] Kromer R, Shafin R, Boelefahr S, Klemm M. An automated approach for localizing retinal blood vessels in confocal scanning laser ophthalmoscopy fundus images. *J Med Biol Eng*. 2016; 36: 485–494. 27688743 10.1007/s40846-016-0152-x PMC5020115

[10] Meyer MI, Costa P, Galdran A, Mendonça AM, Campilho A. A deep neural network for vessel segmentation of scanning laser ophthalmoscopy images. In: *Image Analysis and Recognition: 14th International Conference, ICIAR 2017, Montreal, QC, Canada, July 5–7, 2017, Proceedings 14*. New York, NY: Springer; 2017: 507–515.

[11] Hatamizadeh A, Hosseini H, Patel N, et al. Ravir: a dataset and methodology for the semantic segmentation and quantitative analysis of retinal arteries and veins in infrared reflectance imaging. *IEEE J Biomed Health Inform*. 2022; 26(7): 3272–3283. 35349464 10.1109/JBHI.2022.3163352

[12] Perez-Rovira A, MacGillivray T, Trucco E, et al. Vampire: vessel assessment and measurement platform for images of the retina. In: *2011 Annual International Conference of the IEEE Engineering in Medicine and Biology Society*. *Annu Int Conf* *IEEE* *Eng Med Biol Soc*. 2011; 2011: 3391–3394. 22255067 10.1109/IEMBS.2011.6090918

[13] Zhou Y, Wagner SK, Chia MA, et al. Automorph: automated retinal vascular morphology quantification via a deep learning pipeline. *Transl Vis Sci Technol*. 2022; 11(7): 12. 10.1167/tvst.11.7.12 PMC9290317 35833885

[14] Engelmann J, Villaplana-Velasco A, Storkey A, Bernabeu MO. Robust and efficient computation of retinal fractal dimension through deep approximation. In: *International Workshop on Ophthalmic Medical Image Analysis*. New York, NY: Springer; 2022: 84–93.

[15] Burke J, Engelmann J, Hamid C, et al. An open-source deep learning algorithm for efficient and fully automatic analysis of the choroid in optical coherence tomography. *Transl Vis Sci Technol*. 2023; 12(11): 27. 10.1167/tvst.12.11.27 PMC10668622 37988073

[16] Engelmann J, Burke J, Hamid C, et al. Choroidalyzer: an open-source, end-to-end pipeline for choroidal analysis in optical coherence tomography. *Invest Ophthalmol Vis Sci*. 2024; 65(6): 6. 10.1167/iovs.65.6.6 PMC11156207 38833259

[17] Dhaun N . Optical coherence tomography and nephropathy: the octane study, https://clinicaltrials.gov/ct2/show/NCT02132741, 2014. ClinicalTrials.gov identifier: NCT02132741. Updated November 4, 2022. Accessed May 31, 2023.

[18] Ritchie CW, Ritchie K. The prevent study: a prospective cohort study to identify mid-life biomarkers of late-onset Alzheimer's disease. *BMJ Open*. 2012; 2(6): e001893. 10.1136/bmjopen-2012-001893 PMC3533047 23166135

[19] Ritchie CW, Wells K, Ritchie K. The prevent research programme–a novel research programme to identify and manage midlife risk for dementia: the conceptual framework. *Int Rev Psychiatry*. 2013; 25(6): 748–754. 24423227 10.3109/09540261.2013.869195

[20] Kearns PKA, Martin SJ, Chang J, et al. Futurems cohort profile: a Scottish multicentre inception cohort study of relapsing-remitting multiple sclerosis. *BMJ Open*. 2022; 12(6): e058506. 10.1136/bmjopen-2021-058506 PMC9244691 35768080

[21] Chen Y, Larraz J, Wong M, et al. Longitudinal retinal imaging study of newly diagnosed relapsing-remitting multiple sclerosis in Scottish population: baseline and 12 months follow-up profile of FutureMS retinal imaging cohort. *BMJ Open Ophthalmol*. 2022; 7(1): e001024. 10.1136/bmjophth-2022-001024 PMC9315911 36161838

[22] Cameron JR, Ballerini L, Langan C, et al. Modulation of retinal image vasculature analysis to extend utility and provide secondary value from optical coherence tomography imaging. *J Med Imaging (Bellingham)*. 2016; 3(2): 020501. 27175375 10.1117/1.JMI.3.2.020501 PMC4852212

[23] Yushkevich PA, Piven J, Hazlett HC, et al. User-guided 3D active contour segmentation of anatomical structures: significantly improved efficiency and reliability. *Neuroimage*. 2006; 31(3): 1116–1128. 16545965 10.1016/j.neuroimage.2006.01.015

[24] Forrester JV, Dick AD, McMenamin PG, Roberts F, Pearlman E. Chapter 1 - anatomy of the eye and orbit. In: Forrester JV, Dick AD, McMenamin PG, Roberts F, Pearlman E, eds. *The Eye*. 4th ed. St. Louis, MO: W.B. Saunders; 2016: 1–102.e2.

[25] Iakubovskii P . Segmentation models pytorch, https://github.com/qubvel/segmentation_models.pytorch, 2019.

[26] Cardoso MJ, Li W, Brown R, et al. Monai: an open-source framework for deep learning in healthcare. *arXiv preprint arXiv:2211.02701*, 2022. Available at: 10.48550/arXiv.2211.02701.

[27] Threlfall A, Gibbon S, Cameron J, MacGillivray T. A publicly available vessel segmentation algorithm for SLO images. *arXiv preprint arXiv:2311.17525*, 2023. Available at: 10.48550/arXiv.2311.17525.

[28] Kingma DP, Ba J. Adam: a method for stochastic optimization. *arXiv preprint arXiv:1412.6980*, 2014. Available at: 10.48550/arXiv.1412.6980.

[29] Howard A, Sandler M, Chu G, et al. Searching for MobileNetV3. In: *Proceedings of the IEEE/CVF International Conference on Computer Vision*. 2019: 1314–1324. *arXiv preprint arXiv:1905.02244*, 2014. Available at: 10.48550/arXiv.1905.02244.

[30] Deng J, Dong W, Socher R, Li Li-J, Li K, Fei-Fei Li. ImageNet: a large-scale hierarchical image database. In: *2009 IEEE Conference on Computer Vision and Pattern Recognition*. IEEE; 2009: 248–255.

[31] Loshchilov I, Hutter F. Decoupled weight decay regularization. *arXiv preprint arXiv:1711.05101*, 2017. Available at: 10.48550/arXiv.1711.05101.

[32] He K, Zhang X, Ren S, Sun J. Deep residual learning for image recognition. In: *Proceedings of the IEEE conference on computer vision and pattern recognition*. 2016: 770–778. Available at: https://ieeexplore.ieee.org/document/7780459.

[33] Falconer K . *Fractal geometry: mathematical foundations and applications*. Hoboken, NY: John Wiley & Sons; 2004.

[34] Grisan E, Foracchia M, Ruggeri A. A novel method for the automatic evaluation of retinal vessel tortuosity. In: *Proceedings of the 25th Annual International Conference of the IEEE Engineering in Medicine and Biology Society (IEEE Cat. No. 03CH37439)*. IEEE; 2003:1: 866–869.

[35] Knudtson MD, Lee KE, Hubbard LD, Wong TY, Klein R, Klein BEK. Revised formulas for summarizing retinal vessel diameters. *Curr Eye Res*. 2003; 27(3): 143–149. 14562179 10.1076/ceyr.27.3.143.16049

[36] Martin Bland J, Altman DG. Statistical methods for assessing agreement between two methods of clinical measurement. *Lancet*. 1986; 327(8476): 307–310. 2868172

[37] Engelmann J, Moukaddem D, Gago L, Strang N, Bernabeu MO. Applicability of Oculomics for individual risk prediction: repeatability and robustness of retinal fractal dimension using DART and AutoMorph. *Invest Ophthalmol Vis Sci*. 2024; 65(6): 10. 10.1167/iovs.65.6.10 PMC11160956 38842831

[38] Hoffmann EM, Zangwill LM, Crowston JG, Weinreb RN. Optic disk size and glaucoma. *Surv Ophthalmol*. 2007; 52(1): 32–49. 17212989 10.1016/j.survophthal.2006.10.002 PMC1850981

[39] Mishra A, Pattnaik L, Mishra S, Kumar Panigrahi P, Mohanty S. Assessment of changes in optic disc parameters and peripapillary retinal nerve fiber layer thickness in myopic patients and its correlation with axial length and degree of myopia. *Indian J Ophthalmol*. 2022; 70(12): 4343–4348. 36453342 10.4103/ijo.IJO_1229_22 PMC9940592

[40] Brautaset R, Birkeldh U, Alstig PF, Wiken P, Nilsson M. Repeatability using automatic tracing with Canon OCT-HS100 and Zeiss Cirrus HD-OCT 5000. *PLoS One*. 2016; 11(2): e0149138. 26867021 10.1371/journal.pone.0149138 PMC4750906

[41] Garg G, Venkatesh P, Chawla R, Takkar B, Temkar S, Damodaran S. Normative data of retinal arteriolar and venular calibre measurements determined using confocal scanning laser ophthalmoscopy system–importance and implications for study of cardiometabolic disorders. *Indian J Ophthalmol*. 2022; 70(5): 1657–1663. 35502046 10.4103/ijo.IJO_2162_21 PMC9333006

[42] Wagner SK, Fu DJ, Faes L, et al. Insights into systemic disease through retinal imaging-based Oculomics. *Transl Vis Sci Technol*. 2020; 9(2): 6. 10.1167/tvst.9.2.6 PMC7343674 32704412

[43] Kamran Ikram M, Ong YiT, Cheung CY, Wong TY. Retinal vascular caliber measurements: clinical significance, current knowledge and future perspectives. *Ophthalmologica*. 2013; 229(3): 125–136. 23006932 10.1159/000342158

[44] McGrory S, Taylor AM, Pellegrini E, et al. Towards standardization of quantitative retinal vascular parameters: comparison of SIVA and VAMPIRE measurements in the Lothian Birth Cohort 1936. *Transl Vis Sci Technol*. 2018; 7(2): 12. 10.1167/tvst.7.2.12 PMC5868859 29600120

[45] Sodhi SK, Golding J, Trimboli C, Choudhry N. Feasibility of peripheral oct imaging using a novel integrated SLO ultra-widefield imaging sweptsource oct device. *Int Ophthalmol*. 2021; 41: 2805–2815. 33830372 10.1007/s10792-021-01837-7 PMC8289804

[46] Zhang Z, Li M, Sun Y, Wei Y, Zhang S. Multicolor scanning laser ophthalmoscopy strengthens surgeons’ preoperative decision-making and intraoperative performance on epiretinal membrane. *Transl Vis Sci Technology*. 2020; 9(13): 36. 10.1167/tvst.9.13.36 PMC7757626 33384890

