# Supplementary material for: SLOctolyzer: Fully Automatic Analysis Toolkit for Segmentation and Feature Extracting in Scanning Laser Ophthalmoscopy Images
Source: Transl Vis Sci Technol. 2024 Nov 8;13(11):7. doi: 10.1167/tvst.13.11.7 (PMC11552063; doi:10.1167/tvst.13.11.7)
Supplement: Supplement 1 [file tvst-13-11-7_s001.docx]

**Supplementary materials for “SLOctolyzer: Fully automatic analysis toolkit for segmentation and feature extracting in scanning laser ophthalmoscopy images”**

Jamie Burke^1,2^, Samuel Gibbon^2^, Justin Engelmann^3,4^, Adam Threlfall^2^, Ylenia Giarratano^3^, Charlene Hamid^5^, Stuart King^1^, Ian J.C. MacCormick^6,7^ and Tom MacGillivray^2,5,7^

^1^School of Mathematics, University of Edinburgh, Edinburgh, UK

^2^Robert O Curle Ophthalmology Suite, Institute for Regeneration and Repair, University of Edinburgh, UK

^3^Centre for Medical Informatics, University of Edinburgh, Edinburgh, UK

^4^School of Informatics, University of Edinburgh, Edinburgh, UK

^5^Clinical Research Facility and Imaging, University of Edinburgh, Edinburgh, UK

^6^Institute for Adaptive and Neural Computation, School of Informatics, University of Edinburgh, Edinburgh, UK

^7^Centre for Clinical Brain Sciences, University of Edinburgh, Edinburgh, UK

^∗^Corresponding and lead author;

Email address: Jamie.Burke@ed.ac.uk

**Abstract**

**Purpose** : The purpose of this study was to describe the SLOctolyzer: an open-source analysis toolkit for en face retinal vessels in infrared reflectance scanning laser ophthalmoscopy (SLO) images.

**Methods** : The SLOctolyzer includes two main modules: segmentation and measurement. The segmentation module uses deep learning methods to delineate retinal anatomy, and detects the fovea and optic disc, whereas the measurement module quantifies the complexity, density, tortuosity, and caliber of the segmented retinal vessels. We evaluated the segmentation module using unseen data and measured its reproducibility.

**Results** : The SLOctolyzer’s segmentation module performed well against unseen internal test data (Dice for all-vessels = 0.91; arteries =

0.84; veins = 0.85; optic disc = 0.94; and fovea = 0.88). External validation against severe retinal pathology showed decreased performance (Dice for arteries = 0.72; veins = 0.75; and optic disc = 0.90). The SLOctolyzer had good reproducibility (mean difference for fractal dimension = – 0.001; density = –0.0003; caliber = –0.32 microns; and tortuosity density = 0.001). The SLOctolyzer can process a 768 × 768 pixel macula-centered SLO image in under 20 seconds and a disc-centered SLO image in under 30 seconds using a laptop CPU.

**Conclusions** : To our knowledge, the SLOctolyzer is the first open-source tool to convert raw SLO images into reproducible and clinically meaningful retinal vascular parameters. It requires no specialist knowledge or proprietary software, and allows manual correction of segmentations and re-computing of vascular metrics. The SLOctolyzer is freely available at [https://github.com/jaburke166/SLOctolyzer.](https://github.com/jaburke166/SLOctolyzer)

**Translational Relevance** : SLO images are captured simultaneous to optical coherence tomography (OCT), and we believe our software will be useful for extracting retinal vascular measurements from large OCT image sets and linking them to ocular or systemic diseases.

1

# Simultaneous OCT + SLO capture during acquisition

Supplementary Fig. S1 shows a screenshot from the Heidelberg Eye Explorer (HEYEX) software (version 1.12.1.0) (Heidelberg Engineering, Heidelberg, Germany) of an OCT volume for an individual’s left eye. During OCT capture, the confocal SLO image (left) is used to reference the location of the B-scans (right) captured during acquisition. This is advantageous particularly for registering follow-up scans.


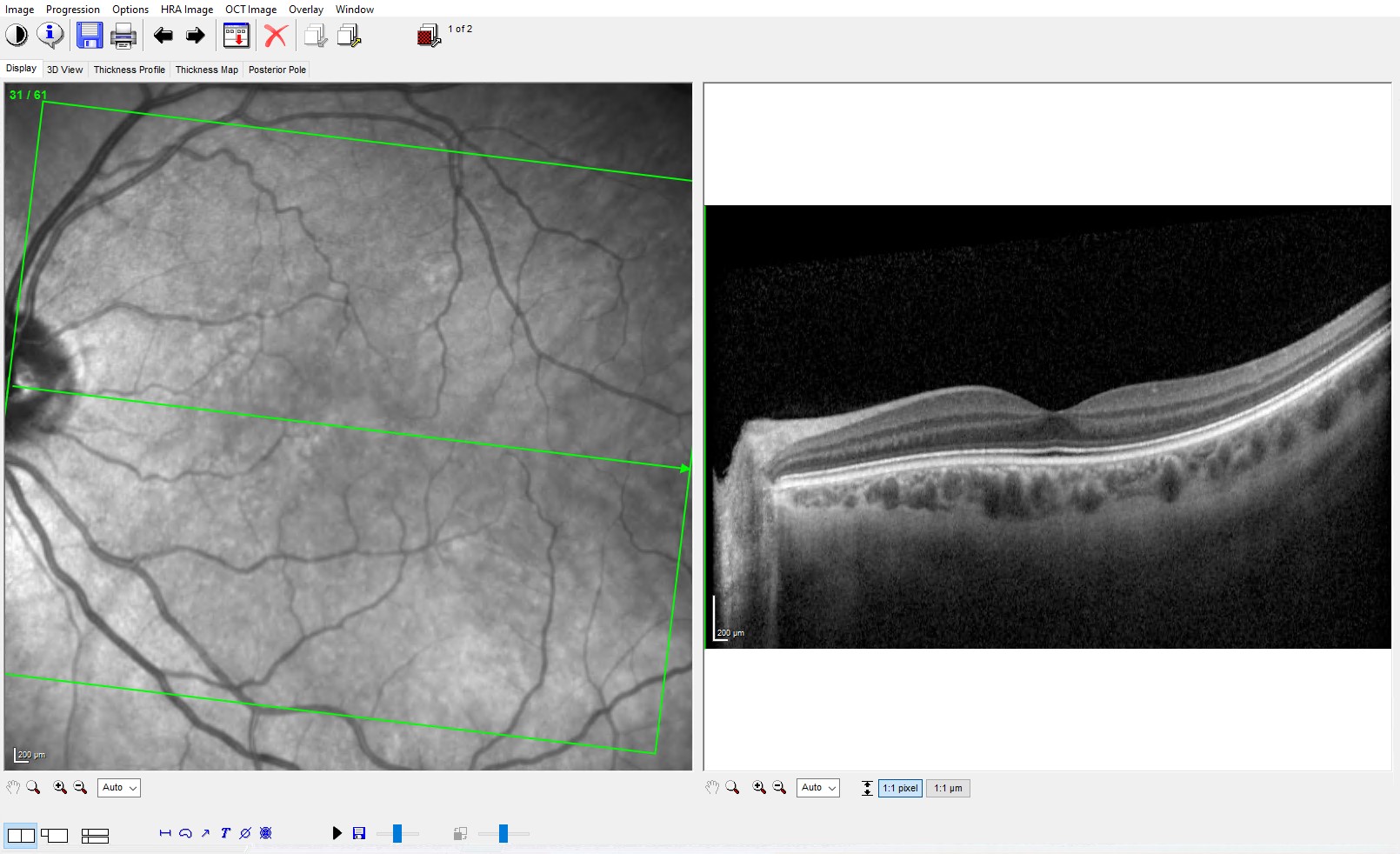


**Figure S1** Screenshot from Heidelberg Eye Explorer (HEYEX) of an OCT volume capture during acquisition. (Left) the confocal SLO image with the acquisition location of the OCT volume overlaid in green. (Right) the corresponding fovea-centred OCT B-scan of the OCT volume.

# SLOctolyzer’s training data for artery-vein-optic disc detection

Supplementary Fig. S2 shows some exemplary SLO images selected from the i-Test and FutureMS cohorts for building the artery-veinoptic disc segmentation model. The total set of 30 SLO images were selected to provide a variety of image-features during training and evaluation, such as blur, non-uniform illumination, and contrast artefacts, as well as abnormal retinal features such as vessel tortuosity or optic nerve head atrophy.


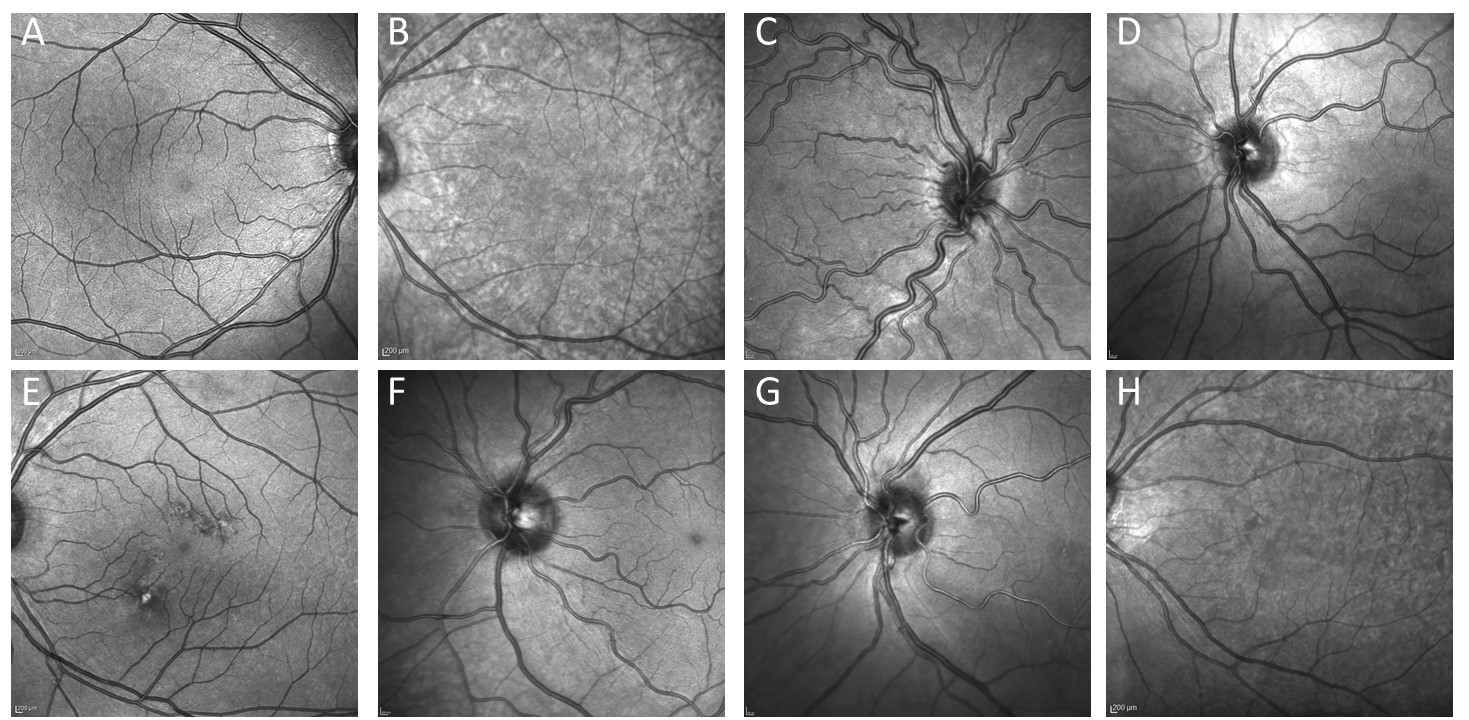


**Figure S2** Selection of the data used to build the artery-vein-optic disc segmentation model, highlighting features of interest in the image which are advantageous for diverse training, such as blur (A), contrast artefacts (B,E,H), non-uniform illumination (D, F, G), vessel tortuosity (C) and optic nerve head atrophy (H).

**Manual detection of the fovea**

# Examples of detecting the fovea

As part of the manual detection of the fovea coordinate on SLO images, author J.B. manually selected the fovea coordinate for all cohorts but the i-Test one.

For en face SLO fovea detection the pixel coordinate representing the fovea pit was detected as the centre of a small, dark and circular region representing the depression on the retinal tissue (sometimes showing a hyperreflective spot at the centre of this region, representing the foveal pit). This is typically seen in the centre of the macula-centred SLO image, and at the centre-row but skewed to the far right- or left-column for disc-centred images, depending on the eye’s laterality. To aid detection, the small arterioles and venules branching from the major vessels were used to guide the centre of the macula and thus the fovea pit for more challenging cases. Supplementary Fig. S3 shows some examples of en face SLO images with their foveal pit identified by red arrows, with supporting text to describe the detection in each case.


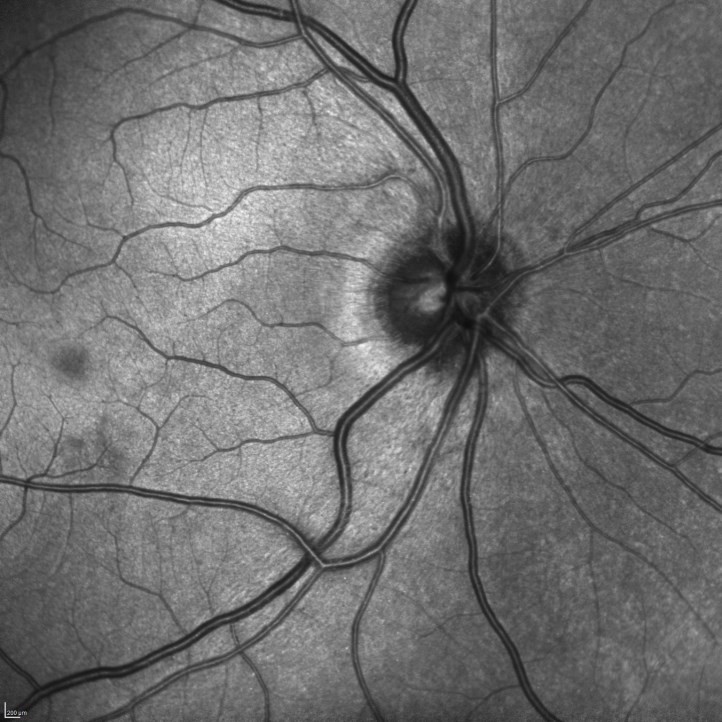

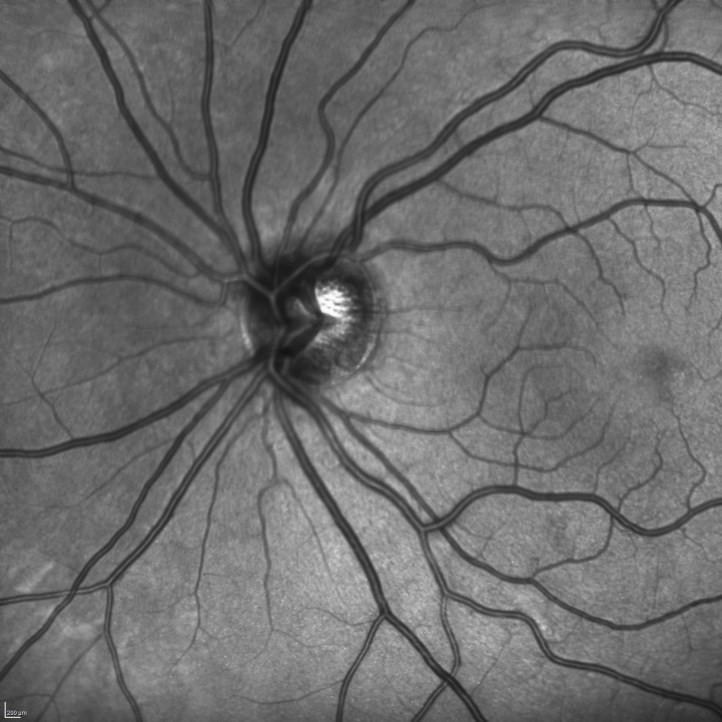

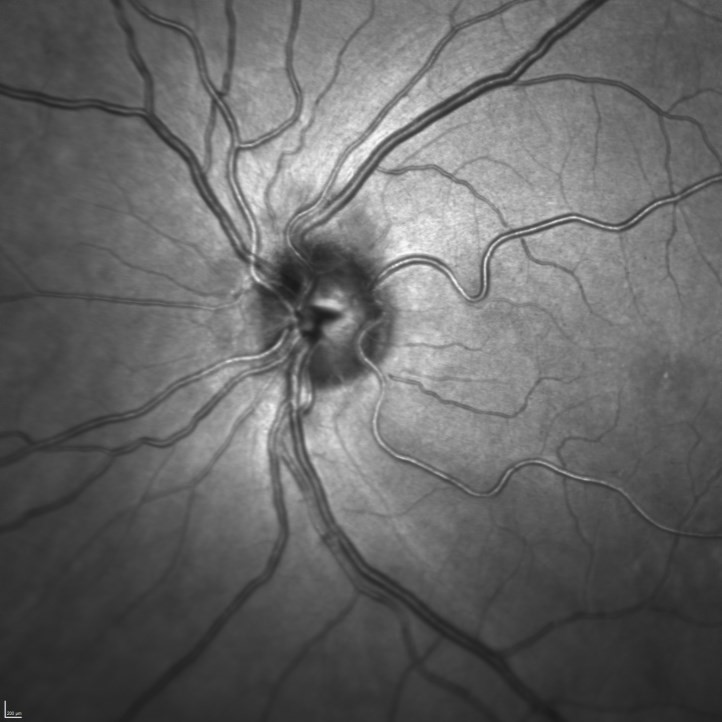

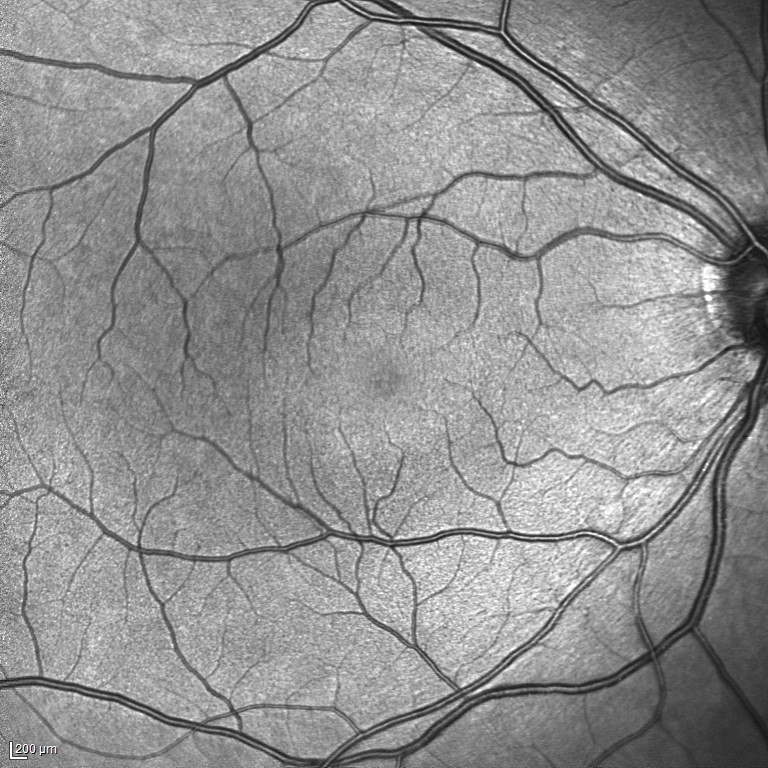

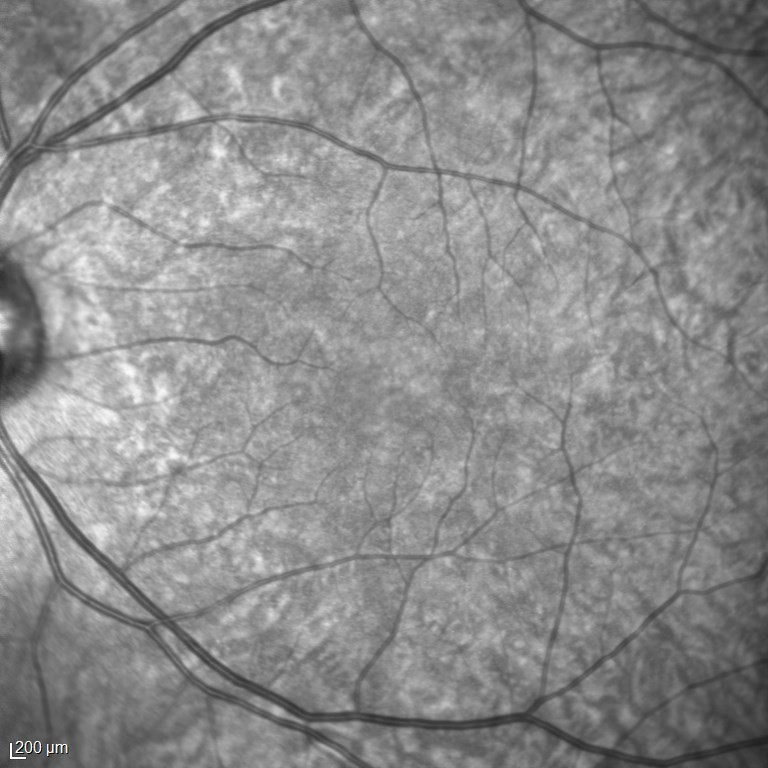

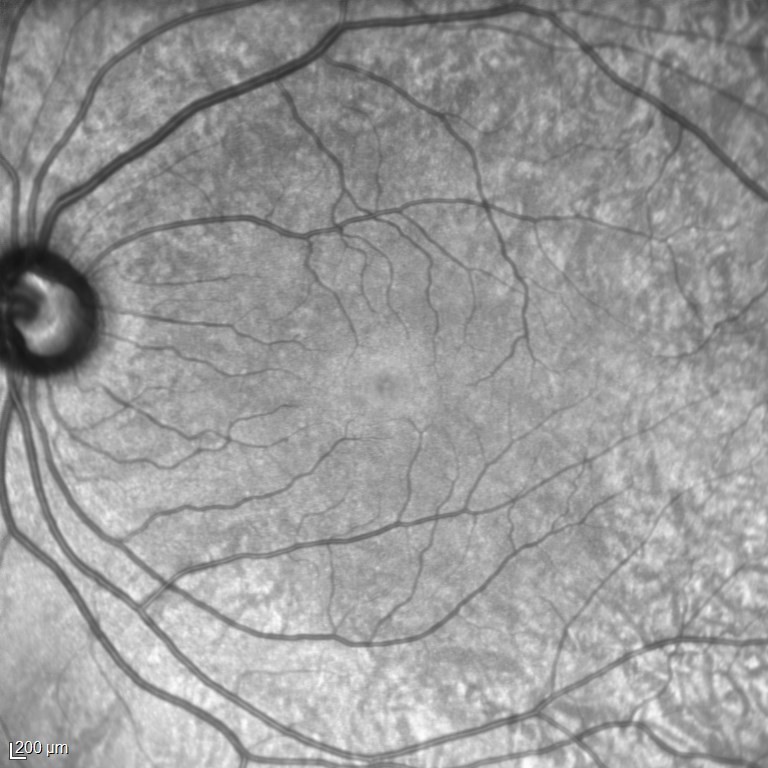


Faint, but visible darker

circular region with

hyperreflective centre.

Obvious, dark circular region.

Obvious, dark circular region.

Faint, but visibly dark

circular region. Arterioles and

Venules help determine centre.

Faint, but visibly dark

circular region. Arterioles and

Venules help determine centre.

No dark spot, but arterioles

Venules help guide approximate

Location of foveola centralis.

**Figure S3** Examples of foveal pit detection for en face SLO images, with text overlaid to describe the detection process in each case.

For a fovea-centred OCT B-scan, we used Choroidalyzer^1^ to define the foveal pit as a single pixel coordinate is at the point of deepest depression in the B-scan. This appears as a dip in the retina with a small hyperreflective region in the centre of this depression, often aligned with a ridge formed at the photoreceptor layer. Fortunately, this detection process is deterministic through Choroidalyzer, and was cross-referenced to the corresponding en face SLO image as we know the transversal position of the fovea on the B-scan and the exact location of acquisition of the B-scan on the SLO image. Supplementary Fig. S4 shows some examples of cross-sectional OCT B-scan with their foveal pit identified by red arrows, with supporting text to describe the detection in each case.


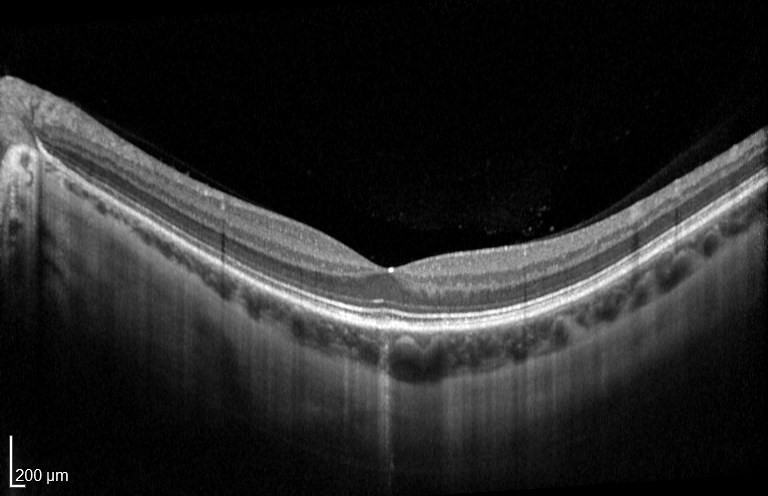

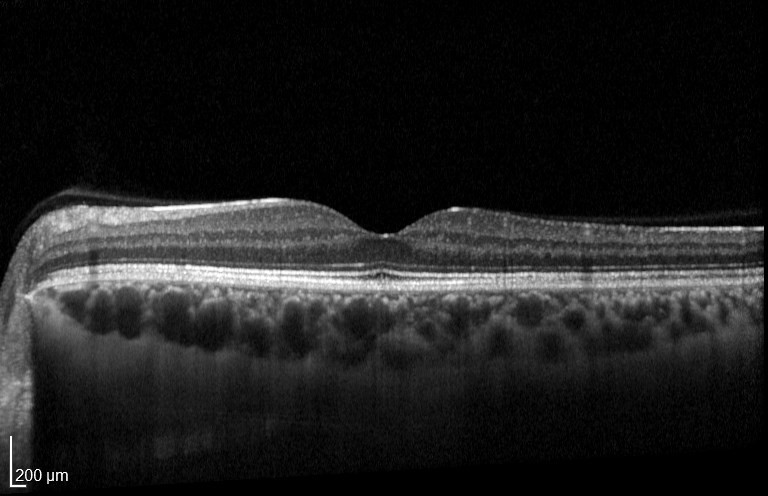

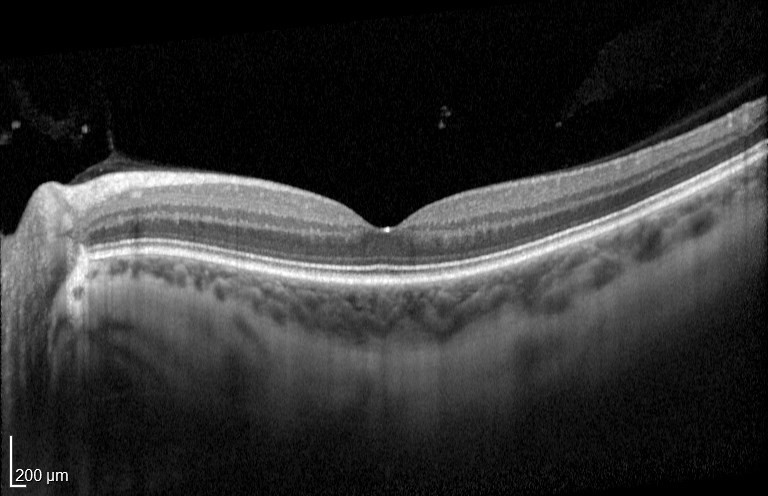

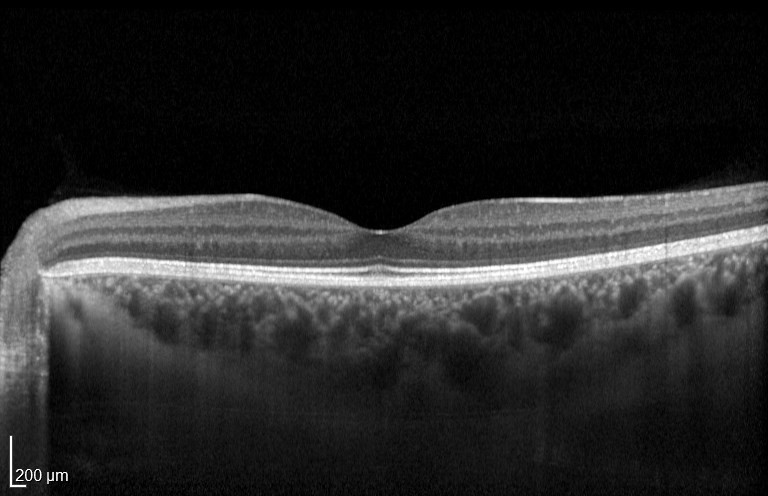

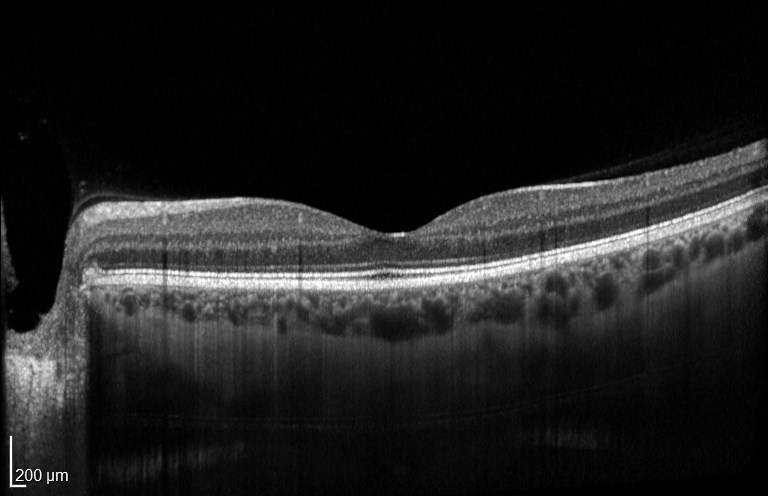

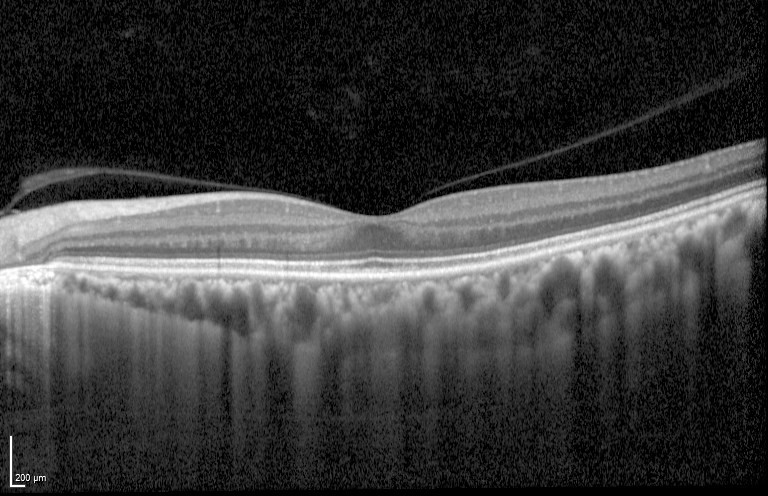


Hyperreflective centre above

o

uter retinal

ridge.

Hyperreflective centre above

o

uter retinal

ridge.

Hyperreflective centre above

o

uter retinal

ridge.

Preference to select hyperreflective

centre when maligned to

outer retinal

ridge.

Guided by outer retinal ridge, and

point of deepest depression.

Hyperreflective centre above

outer retinal ridge.

**Figure S4** Examples of foveal pit detection for fovea-centred, cross-sectional OCT B-scans, with text overlaid to describe the detection process in each case.

# Rater repeatability

As a measure of rater repeatability, author J.B. re-selected the fovea coordinate for all 516 SLO images two months after the initial manual detection, and compared the (*x*, *y*)-positioning using mean absolute error and intra-class correlation. Like the training pipeline for the fovea detection model, fovea segmentation masks were generated for each batch of coordinates and compared using the area under the receiver operating characteristic curve (AUC) and dice coefficient. Evaluation was performed after all images were resized to a common image resolution of 768 × 768 pixels for interpretable comparison.

The repeatability results are listed below in supplementary Table S1. Results for the i-Test cohort represent inter-rater repeatability

(manual detection against accurate ground truth data from cross-referenced OCT B-scan using Choroidalyzer^1^), and results for the remaining four cohorts of data represent intra-rater repeatability. Intra-rater repeatability had an excellent average intra-class correlation of 0.99 and a strong intra-class correlation for inter-rater repeatability of 0.82 was also reported.

AUC Dice ICC(3,1) MAE (px)

|  |  |  | x y | x y |
| --- | --- | --- | --- | --- |
| Others | 0.96 | 0.93 | 0.99 0.99 | 3.39 3.00 |
| i-Test | 0.94 | 0.89 | 0.77 0.87 | 4.75 4.52 |

**Table S1** Repeatability of detecting the fovea on the i-Test dataset (which measure inter-rater repeatability) and the remaining cohorts (which measure intra-rater repeatability). AUC, area under the receiver operating characteristic curve; ICC, intra-class correlation for single fixed raters; MAE, mean absolute error (in pixels)

## Manual annotation of artery-vein-optic disc

The optic disc was classified as both the cup and rim together, and its margin was defined by the sharpest intensity transition between the darker rim and brighter retinal tissue constituting the retinal nerve fiber layer. For accurate artery-vein classification, the list below is a set of classification rules each grader followed:

1. **Alternating** rule: It’s common to see arteries and veins appear on the fundus in an alternative nature, for efficient oxygen transport^2^.
2. **Junction** rule: It is common to observe arteriovenous crossings (a junction of four lanes) and same-class vessel bifurcations (a junction of three lanes). Given a crossing where there are more than three lanes, it’s likely to be an arteriovenous crossing. Otherwise, it is a same-class vessel bifurcation.
3. **Thickness** rule: Veins are typically thicker than arteries^3^.
4. **Brightness** rule: Veins are typically darker than arteries^4^.

Supplementary Fig. S5 shows an exemplary SLO image and annotations to describe each of the rules followed by the graders.


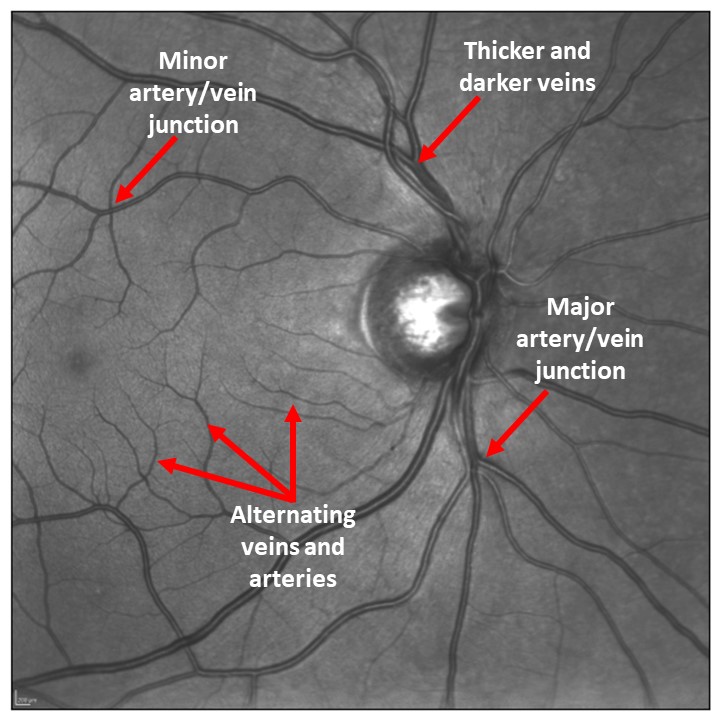


**Figure S5** An exemplary SLO image with annotations to describe each of the four rules for artery-vein classification.

A protocol was created to ensure consistent and comparable segmentations by each grader using ITK-Snap^5^. After application of the binary vessel segmentation model to detect all the vessels in each SLO image, the graders followed the rules listed below during

pixel-level annotation:

1. Given an arteriovenous crossing, select the class which is most obviously overlaid on top the other class. If unsure, select the largest vessel.
2. Do not classify any vessels within the optic disc.
3. Complete any disconnected vessels.
4. Do not extend vessels which have visibly not been segmented.
5. Remove false positive pixels, i.e. retinal tissue which has been classed as vessel.

Supplementary Fig. S6 shows an exemplary SLO image with a binary vessel map overlaid using the binary vessel detection model, with annotations to highlight each of the four segmentation rules each grader followed.


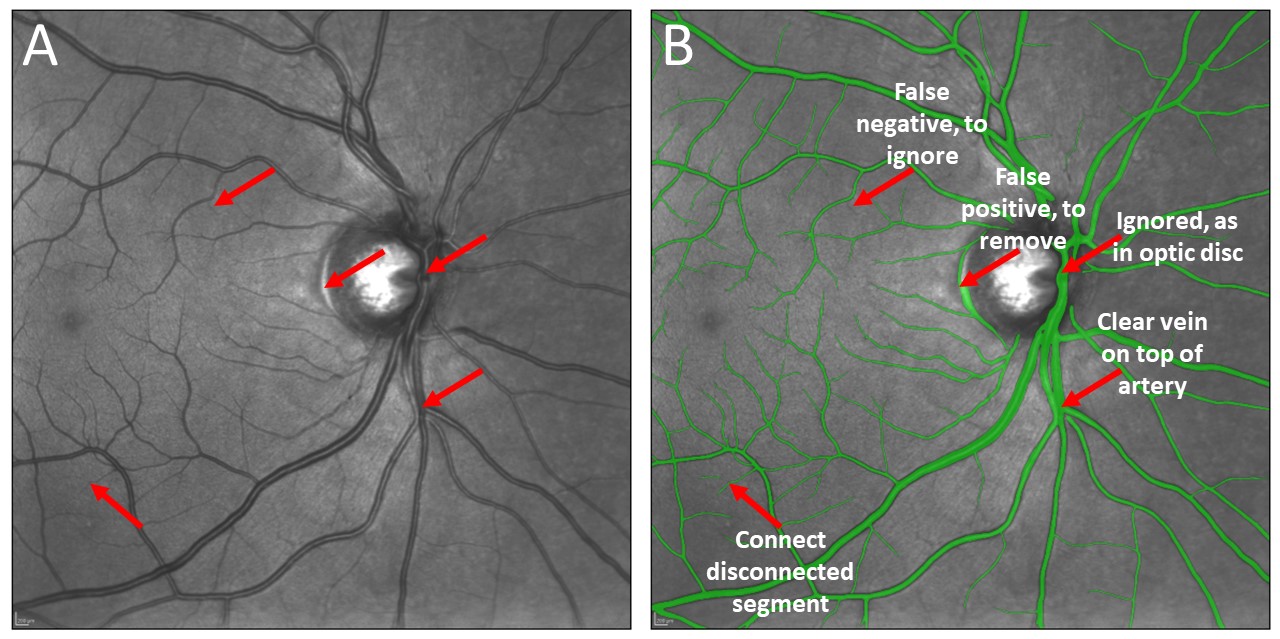


**Figure S6** An exemplary SLO image (A) with a raw binary vessel mask (B) superimposed in green with annotations to describe each segmentation rule followed during annotation.

## Inter-rater agreement of graders for artery-vein-optic disc detection

Supplementary Table S2 shows the results of comparing the segmentations for each task (vessel/artery/vein/optic disc) across the three raters using the dice coefficient (ignoring background). All dice scores were greater than 0.93, suggesting strong agreement between graders.

| Pairwise grader | Vessel | Artery | Vein | Optic disc |
| --- | --- | --- | --- | --- |
| Grader 1 vs. Grader 2 | 0.99 | 0.96 | 0.97 | 0.94 |
| Grader 1 vs. Grader 3 | 0.99 | 0.95 | 0.96 | 0.95 |
| Grader 2 vs. Grader 3 | 0.99 | 0.95 | 0.96 | 0.94 |

**Table S2** Inter-grader agreement between the three segmentation raters using the Dice similarity coefficient.

## Qualitative evaluation of artery and vein ground truth labels

Supplementary Table S3 show the qualitative grading results from the clinical ophthalmologist (I.M.) for the manual annotations. All

SLO images were graded as having ‘good quality’ or above, except for one, and there was no annotation which received a bad or worse rating. Across the image quality categories, a total of 19 SLO images were marked as ‘very good’, 10 as ‘good’ and 1 as ‘okay’. The SLO image and manual annotation which had the poorest score is shown in supplementary Fig. S7. The clinical ophthalmologist identified an arteriole and venule nasal to the optic disc as misclassified (red arrows). This error amounted to a drop in score from ‘good’ to ‘okay’, suggesting that all other images which rated as ‘good’ had significantly smaller miss-classifications, if any.

| SLO Image quality | Artery-vein rating |
| --- | --- |
| Very good (*n*= 10) | VG: 7, G: 3, O: 0, B: 0, VB: 0 |
| Good (*n*= 13) | VG: 7, G: 5, O: 1, B: 0, VB: 0 |
| Okay (*n*= 7) | VG: 5, G: 2, O: 0, B: 0, VB: 0 |

**Table S3** Qualitative adjudication of the manual annotations for artery and vein classification from a clinical ophthalmologist (I.M.). VG, very good; G, good; O, okay; B, bad; VB, very bad.


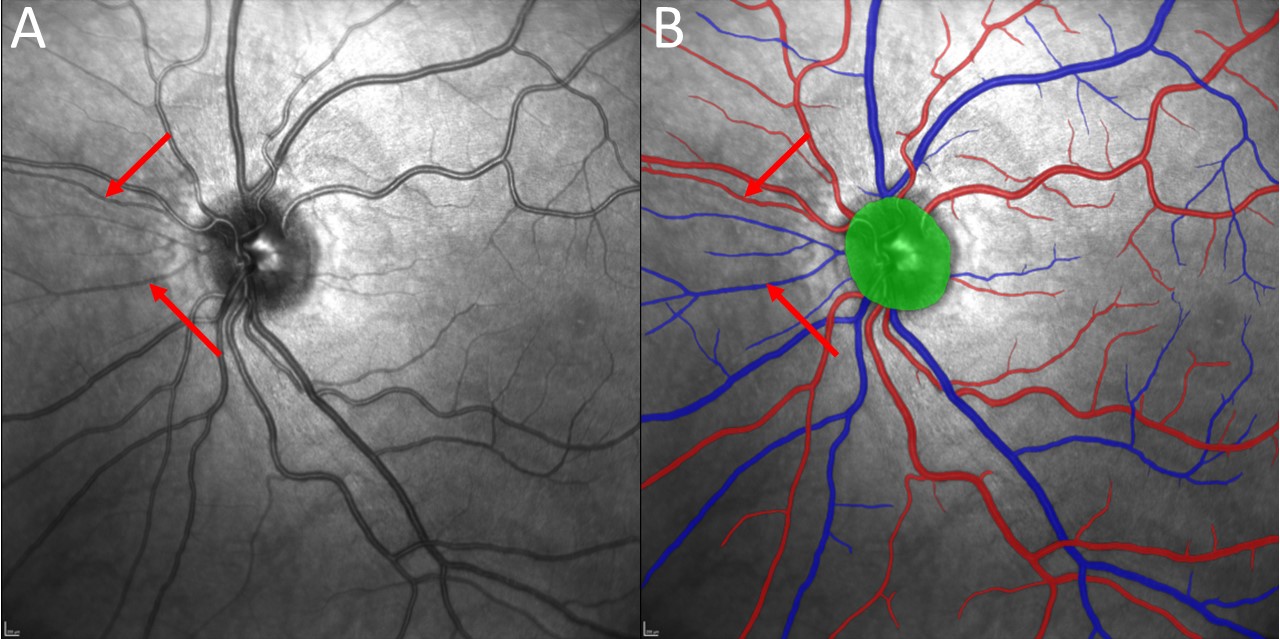


**Figure S7** SLO image (A) and corresponding manual annotation superimposed (B) with poorest score from manual adjudication from clinical ophthalmologist (I.M.). The red arrows indicate where an arteriole/venule was miss-classified.

## RAVIR segmentation label corrections

During the construction of the artery-vein-optic disc detection (AVOD) model, we intended on using the publicly available RAVIR dataset to further enhance and diversify our dataset of SLO images. However, during experimentation, we observed some inconsistent artery and vein classification.

One of our image graders (author J.B.) identified any major errors in the original segmentation labels and corrected them using ITK-Snap^5^. Supplementary Fig. S8 shows an SLO image with the most significant error (A), the SLO image with the original labelling overlaid (B), and the SLO image with the corrected version overlaid (C).

After major corrections were made to all SLO images, we asked a clinical ophthalmologist (author I.M.) to rate the quality of the SLO image, as well as the segmentations before and after correction in a masked and randomised fashion using a 5-point ordinal scale from 2 (very good) to -2 (very bad). We also asked him to rate his preference, with options for both or neither available. Supplementary Table S4 shows the results from this adjudication, stratified by image quality.


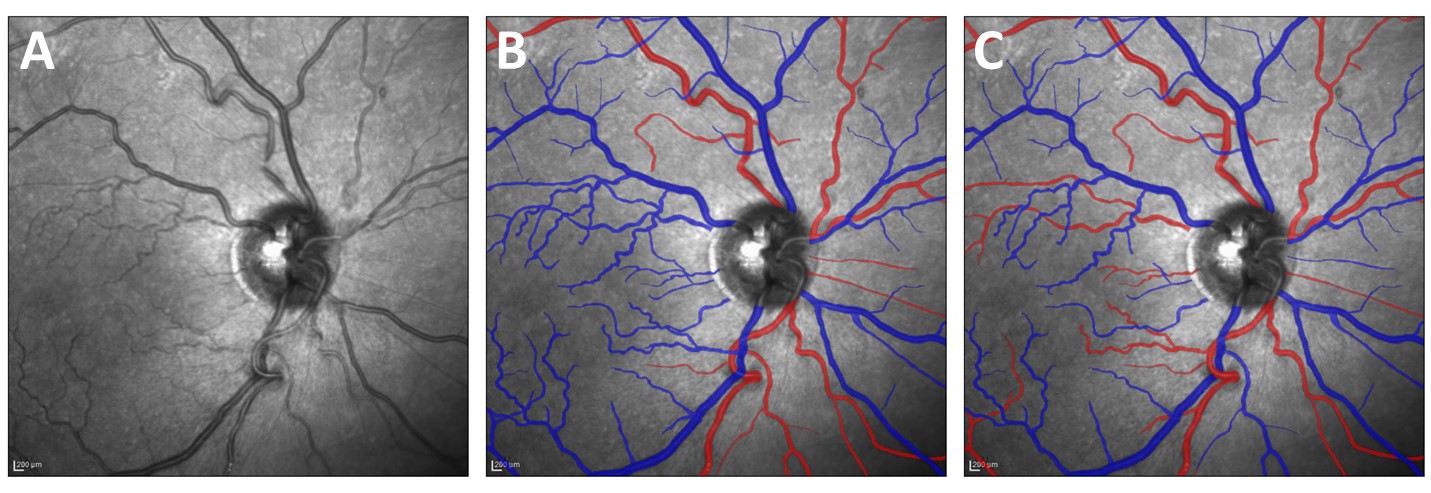


**Figure S8** SLO image (A), original manual annotation superimposed^6^ (B) and corrected manual annotation (C). Here, many of the vessels in the macula were all assumed to be venules in the original annotation.

| Image Quality | Original^6^ | Corrected | Both |
| --- | --- | --- | --- |
| Very good (*N* = 6) | 0 | 3 | 3 |
| Good (*N* = 4) | 0 | 2 | 2 |
| Okay (*N* = 10) | 0 | **7** | 3 |
| Bad (*N* = 3) | 0 | **2** | 1 |
| Total (*N* = 23) | 0 | **14** | 9 |
|  |  |  |  |
| Label | Artery-Vein classification | |  |
| Original^6^ | VG: 6, G: 8, O: 6, B: 2, VB: 1 | |  |
| Corrected | **VG: 10, G: 11, O: 2, B: 0, VB: 0** | |  |

**Table S4** Qualitative adjudication between the original and corrected manual annotations for artery and vein classification in the RAVIR dataset^6^, from a clinical ophthalmologist (I.M.). VG, very good; G, good; O, okay; B, bad; VB, very bad.

We chose to exclude the RAVIR dataset from training the artery-vein-optic disc model and used the corrected segmentation labels as an external test set for our model. We hypothesised this set would pose a significant challenge for our model, which was trained on images related to systemic health.

**SLOctolyzer’s segmentation model architectures**

# Binary vessel detection

For the binary, all-vessel segmentation model, we use a custom UNet deep learning architecture with a depth of 4, as shown in supplementary Fig. S9. A convolution block consists of two 3 × 3 convolution layers, each followed by batch normalisation^7^ and non-linear rectified linear unit activation (ReLU). After an initial convolution block, the model’s DownBlocks double the channel dimension from 48 to 768 while decreasing the spatial dimension by 1/2 to a final, encoded block with a spatial resolution of 20 × 15 with 768 features maps. The model’s UpBlocks reverse the DownBlocks, with each block reducing the channel dimension by 1/2 after applying a 2 × 2 transposed convolution layer with a 2 × 2 stride which scales the spatial dimension by 2. A final 1 × 1 output convolution layer reduces the channel dimension to 1, resulting in the probabilistic segmentation map as output

As described in the main text, offline augmentation extracts 20 random patches of size 320 × 240 pixels from each SLO image

(of native image resolution 768 × 768 pixels) and these are used for training, as shown in the input/output stage of supplementary Fig. S9. At inference, the entire SLO image of image resolution 768 × 768 pixels is fed into the network, outputting a 768 × 768 pixel probability segmentation map.


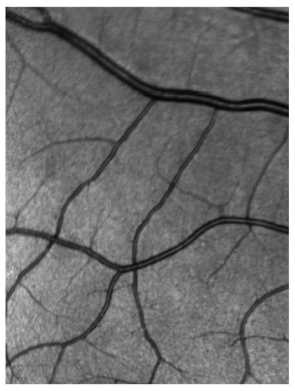

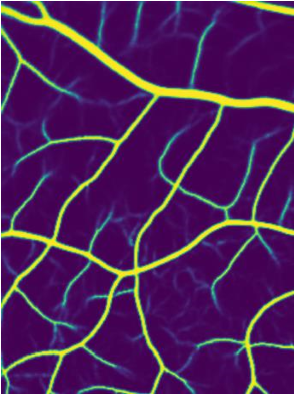

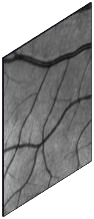

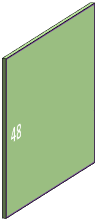


x 320 x

240

1


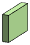


F x M x N

encoder

feature map

All

-

vessel Architecture


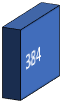

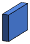


F x M x N

decoder

feature map


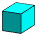


x 20 x 15

768

encoded

block


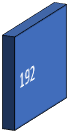

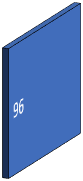


x2 MaxPool2d,

2

2

)

x (

3

x 3 Conv2d,

BatchNorm

,

ReLU

2

x2

ConvTranspose2d

2

)

x (

Skip

connection

1

x1

Conv2d


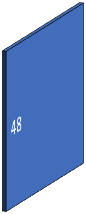

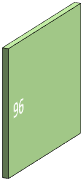

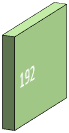

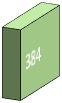

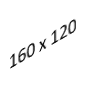

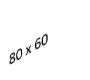

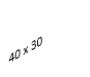

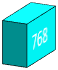


1

240

x 320 x

2

x(

)


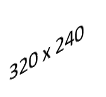


F: Feature maps

M: Number of rows

N: Number of columns


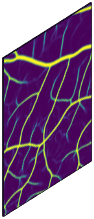


**Figure S9** Custom UNet model architecture for the binary, all-vessel segmentation model.

# Fovea detection

The model used for fovea detection was an ‘off-the-shelf’, pre-trained model from SegmentationModelsPytorch^8^ which utilised a MobileNetV3^9^ backbone encoder arm with a UNet decoder arm (the default setup from SegmentationModelsPytorch). The exact Python code to instantiate the model is seen below, with the SegmentationModelsPytorch package assumed to be installed.

**Code Listing 1** Python code to reproduce model for fovea detection.

| import segmentation_models_pytorch as smp model = smp.Unet(encoder_name="timm-mobilenetv3_small_075",  encoder_weights="imagenet", in_channels=1, classes=1) |
| --- |

# Artery-vein-optic disc detection

The model used for artery-vein-optic disc detection was an ‘off-the-shelf’, pre-trained model from SegmentationModelsPytorch^8^ which utilised a ResNet101^10^ backbone encoder arm with a UNet decoder arm (the default setup from SegmentationModelsPytorch). The exact Python code to instantiate the model is seen below, with the SegmentationModelsPytorch package assumed to be installed.

**Code Listing 2** Python code to reproduce model for artery-vein-optic disc detection.

import segmentation_models_pytorch as smp model = smp.Unet(encoder_name="resnet101", encoder_weights="imagenet", in_channels=1, classes=4)

## Global and local vessel calibre

Measurements of fractal dimension, vessel density and global vessel calibre are not taken across zones B and C because they capture a global measure of the vasculature. Instead, for zones B and C, we compute tortuosity density, central retinal artery/vein equivalents, and local vessel calibre along individual vessel segments in the vessel maps. These vessel segments are defined where crossings and bifurcations are observed.

Supplementary Fig. S10 shows the key differences in global and local vessel calibre. Global vessel calibre is the ratio between vessel pixels and skeletonised vessel pixels (number of yellow pixels divided by brown pixels). This provides a coarse measure of calibre. Local vessel calibre measures the diameter of each vessel segment by traversing it’s length and measuring the diameter of the largest circle which fits entirely within it. A vessel segments’ calibre is then the average value of the list of circle diameters collected as the vessel segment is traversed. Local vessel calibre is thus the overall average of all vessel segment calibres, hence a more granular approach to assess vessel calibre.


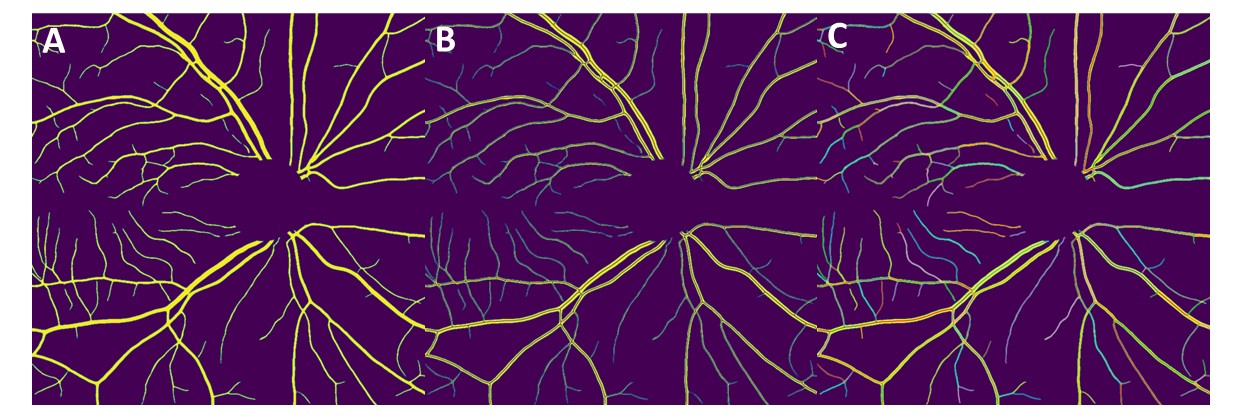


**Figure S10** (A) SLO binary vessel map. Binary vessel map with skeleton overlaid in brown (B) and individual vessel segments overlaid in an array of colours (C). Note that the optic disc area is removed from the vessel map during feature measurement.

## SLOctolyzer’s interface

Supplementary Fig. S11 shows the core steps to run the SLOctolyzer pipeline. SLOctolyzer can be run either from an integrated development environment (IDE), such as Jupyter, Spyder or Visual Studio Code, or directly from the terminal. All that is required is the input/output directories in a simple configuration text file. A process log for the user is outputted as the pipeline processes each image in turn, utilising GPU acceleration if available. When inputting image files, an optional spreadsheet with additional metadata such as the eye laterality, location and transversal spatial sampling length-scale (in microns-per-pixel) can be provided, so that measurements in pixel units can be converted into microns. For .vol this is not necessary as SLOctolyzer can access the file metadata.


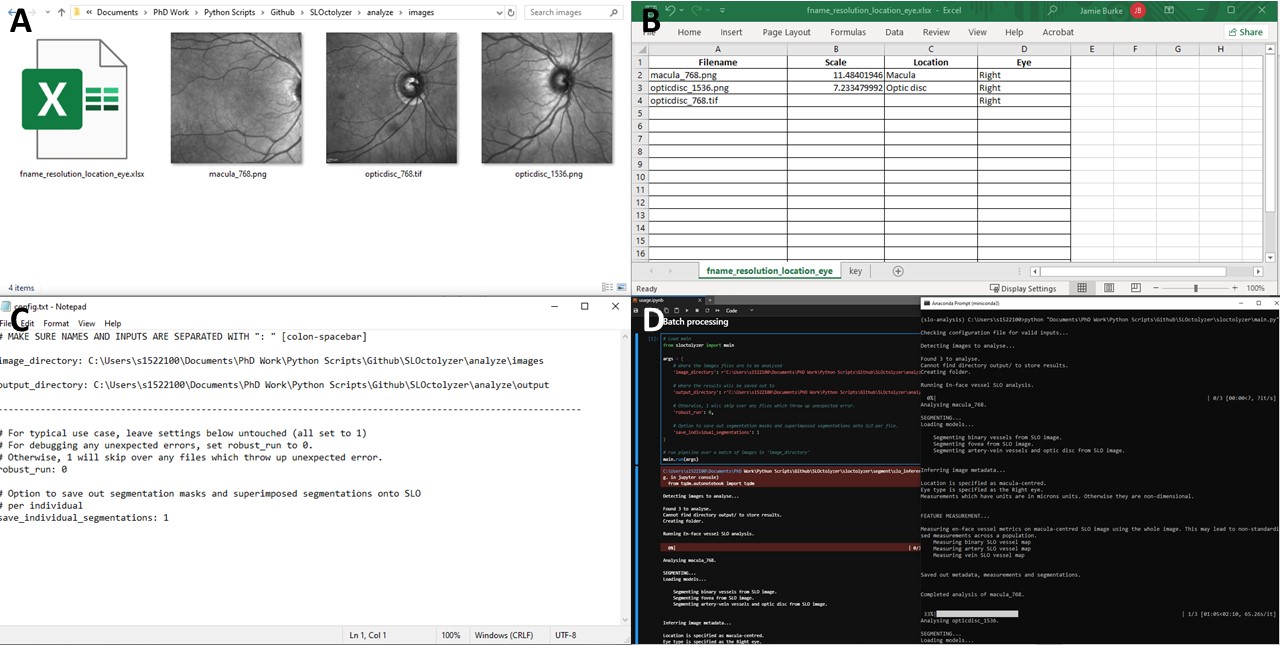


**Figure S11** Summary of the process of running SLOctolyzer on a batch (*n* = 3) of images. (A) Directory of input SLO images to be analysed, with an optional spreadsheet which allows manual data entry of transversal spatial sampling length-scale, laterality and location. (B) Spreadsheet with optional metadata to accompany SLO images during pipeline. Note that missing information is supported. (C) Configuration file specifying the file paths to images and where results will be saved. (D) Demonstration of running SLOctolyzer via JupyterLab or via the terminal.

Supplementary Fig. S12 summarises the output from running SLOctolyzer on a batch (*n* = 3) of images. A folder is generated for each SLO image file analysed, and within each folder contains the segmentations masks, feature measurements and a composite image showing the segmentations superimposed onto the SLO. For ease of assessing segmentation quality, composite segmentation images are saved out for every SLO image analysed into a separate folder. Finally, an output spreadsheet collates all feature measurements for every image analysed row-wise, which can be quickly loaded into a statistical programming language for data analysis.


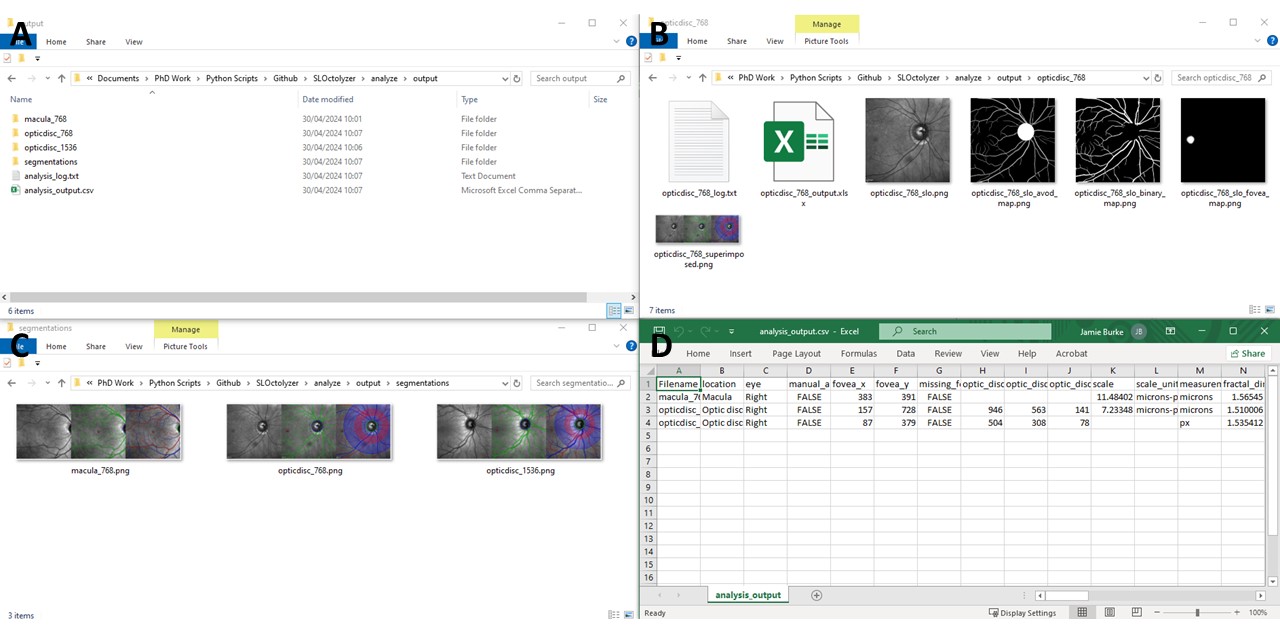


**Figure S12** Summary of the output of running SLOctolyzer on a batch (*n* = 3) of images. (A) Directory of output folders for each of the three image files analysed. (B) Output files for the ‘opticdisc_768’ exemplar SLO image, including the original SLO image, segmentation masks, composite image with segmentations superimposed and a results file with metadata and feature measurements. (C) A directory with composite images of SLO and segmentation masks per image file for quick inspection of segmentation quality. (D) A composite results file with the metadata and features of each image file processed, stored row-wise to facilitate downstream statistical analysis.

SLOctolyzer: Fully Automatic SLO Image Analysis

## Examples of poor performance from reproducibility analysis

Supplementary Fig. S13 shows two examples which had the second largest outliers for tortuosity density and large-vessel calibre

(AVR/CRAE/CRVE). In supplementary Fig. S13(A), while there is no qualitative difference in the binary vessel segmentation, the error in tortuosity density is quite large. This is likely due to vessel disconnectedness between the segmentations (purple arrows).

Nevertheless, the local vessel calibre and fractal dimension residuals are very low.

Additionally, due to the unregistered nature of the SLO image pairs, minor translations/scales can have an inordinate impact on large-vessel calibre reproducibility given the major arteries and veins appear toward the edge of macula-centred SLO images. In supplementary Fig. S13(B), this particular repeated set of SLO images are off by a horizontal translation while intersecting the disc and thus cuts off one of the major arteries and veins (red arrows). This had a significant consequence to the CRVE and CRAE residuals, causing large error. Nevertheless, local vessel calibre remains stable due to taking into account the whole vasculature of the image.


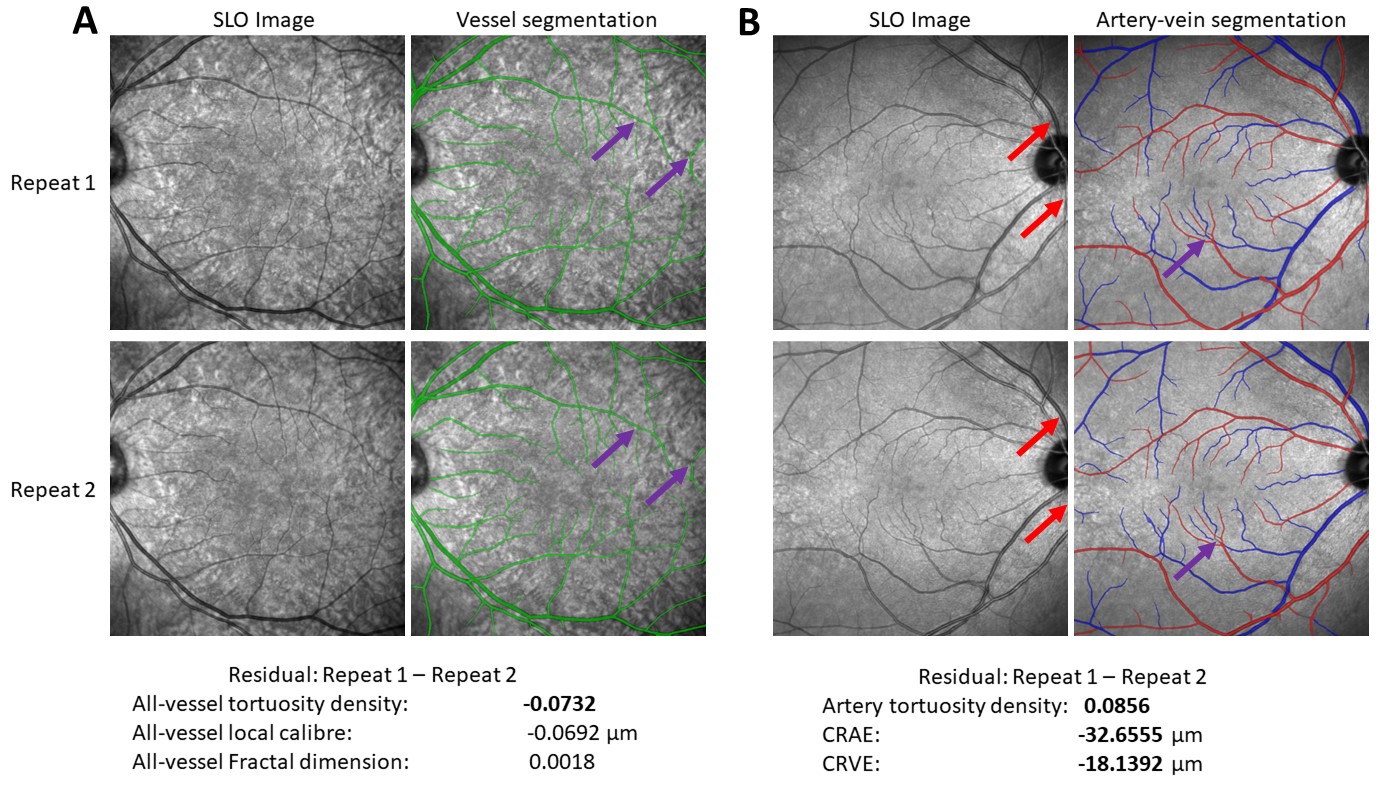


**Figure S13** Some examples of poor agreement in repeated SLO images for tortuosity density (A), and vessel width measurements (B). In Fig. 6 of the main manuscript, (A) is represented by the blue star, while (B) is represented by the red stars.

## References

1. Justin Engelmann, Jamie Burke, Charlene Hamid, Megan Reid-Schachter, Dan Pugh, Neeraj Dhaun, Diana Moukaddem, Lyle Gray, Niall Strang, Paul McGraw, et al. Choroidalyzer: An open-source, end-to-end pipeline for choroidal analysis in optical coherence tomography. *Investigative Ophthalmology & Visual Science*, 65(6):6–6, 2024.
2. Akihiro Ishibazawa, Nihaal Mehta, Osama Sorour, Phillip Braun, Sarah Martin, A Yasin Alibhai, Adnan Saifuddin, Malvika Arya, Caroline R Baumal, Jay S Duker, et al. Accuracy and reliability in differentiating retinal arteries and veins using widefield en face oct angiography. *Translational Vision Science & Technology*, 8(3):60–60, 2019.
3. Gaurav Garg, Pradeep Venkatesh, Rohan Chawla, Brijesh Takkar, Shreyas Temkar, and Sourav Damodaran. Normative data of retinal arteriolar and venular calibre measurements determined using confocal scanning laser ophthalmoscopy system–importance and implications for study of cardiometabolic disorders. *Indian Journal of Ophthalmology*, 70(5):1657–1663, 2022.
4. Olaf Brinchmann-Hansen and Leiv Sandvik. The intensity of the light reflex on retinal arteries and veins. *Acta Ophthalmologica*, 64(5):547–552, 1986.
5. Paul A. Yushkevich, Joseph Piven, Heather Cody Hazlett, Rachel Gimpel Smith, Sean Ho, James C. Gee, and Guido Gerig. Userguided 3D active contour segmentation of anatomical structures: Significantly improved efficiency and reliability. *Neuroimage*, 31(3):1116–1128, 2006.
6. Ali Hatamizadeh, Hamid Hosseini, Niraj Patel, Jinseo Choi, Cameron C Pole, Cory M Hoeferlin, Steven D Schwartz, and Demetri Terzopoulos. Ravir: A dataset and methodology for the semantic segmentation and quantitative analysis of retinal arteries and veins in infrared reflectance imaging. *IEEE Journal of Biomedical and Health Informatics*, 26(7):3272–3283, 2022.
7. Sergey Ioffe and Christian Szegedy. Batch normalization: Accelerating deep network training by reducing internal covariate shift. In *International conference on machine learning*, pages 448–456. pmlr, 2015.
8. Pavel Iakubovskii. Segmentation models pytorch. [https://github.com/qubvel/segmentation_models.pytorch,](https://github.com/qubvel/segmentation_models.pytorch) 2019.
9. Andrew Howard, Mark Sandler, Grace Chu, Liang-Chieh Chen, Bo Chen, Mingxing Tan, Weijun Wang, Yukun Zhu, Ruoming Pang, Vijay Vasudevan, et al. Searching for mobilenetv3. In *Proceedings of the IEEE/CVF international conference on computer vision*, pages 1314–1324, 2019.
10. Kaiming He, Xiangyu Zhang, Shaoqing Ren, and Jian Sun. Deep residual learning for image recognition. In *Proceedings of the IEEE conference on computer vision and pattern recognition*, pages 770–778, 2016.
